# Ice sheets matter for the global carbon cycle

**DOI:** 10.1038/s41467-019-11394-4

**Published:** 2019-08-15

**Authors:** J. L. Wadham, J. R. Hawkings, L. Tarasov, L. J. Gregoire, R. G. M. Spencer, M. Gutjahr, A. Ridgwell, K. E. Kohfeld

**Affiliations:** 1 University of Bristol, Bristol, BS8 1TH UK; 2 National High Magnetic Field Lab and Earth, Ocean and Atmospheric Sciences, Florida State University, Tallahassee, FL 32306 USA; 3 German Research Centre for Geosciences GFZ, 14473 Potsdam, Germany; 4 Memorial University, St. John’s, NF A1B 3X9 Canada; 5 University of Leeds, Leeds, LS6 1AN UK; 6 GEOMAR, 24148 Kiel, Germany; 7 University of California, Riverside, CA 94720 USA; 8 Simon Fraser University, Burnaby, BC 8888 Canada

**Keywords:** Cryospheric science, Biogeochemistry, Carbon cycle

## Abstract

The cycling of carbon on Earth exerts a fundamental influence upon the greenhouse gas content of the atmosphere, and hence global climate over millennia. Until recently, ice sheets were viewed as inert components of this cycle and largely disregarded in global models. Research in the past decade has transformed this view, demonstrating the existence of uniquely adapted microbial communities, high rates of biogeochemical/physical weathering in ice sheets and storage and cycling of organic carbon (>10^4^ Pg C) and nutrients. Here we assess the active role of ice sheets in the global carbon cycle and potential ramifications of enhanced melt and ice discharge in a warming world.

## Abstract (web summary)

Ice sheets have long been overlooked as regulators of the global carbon cycle. In this Review, Wadham and colleagues show how the growth and retreat of ice sheets support the productivity of the oceans and variably store or release organic carbon–in effect, these frozen landscapes must be considered in future assessments of climate impacts on biogeochemical cycling.

## Introduction

**Fig. 1:**
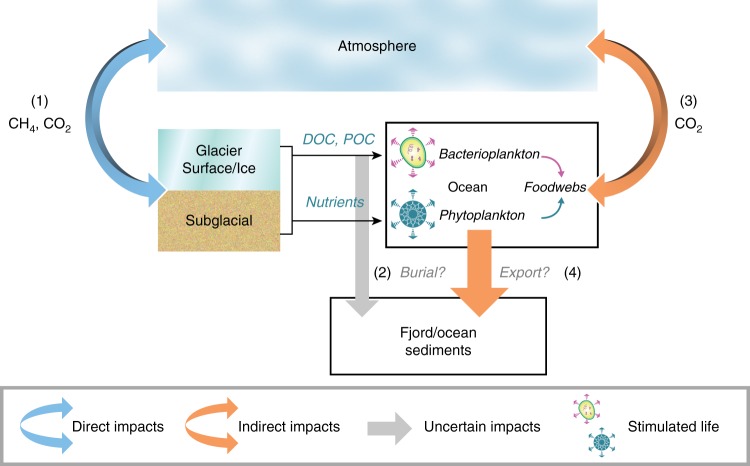
Conceptual diagram of ice sheets in the global carbon cycle. The diagram indicates the potential direct and indirect impacts of ice sheets. (1) direct sequestration or emission of CO_2_/CH_4_ by microbial activity in ice sheets, (2) burial of terrestrial OM exported from ice sheets by rivers, (3) emission of CO_2_ by respiration or uptake by primary production in the oceans (4) export production–OM produced by primary production which is not recycled before it sinks to the ocean floor

For a component of the Earth’s system to impact the global carbon cycle, and potentially influence atmospheric concentrations of carbon dioxide or methane, it must either directly sequester and/or release carbon, or indirectly influence carbon uptake/release in other parts of the Earth system. The notion that ice sheets contain significant carbon stores has its roots in the early 2000s–the great ice sheets were hypothesised to advance over soil and vegetation carbon during glacial periods, with this fossil carbon being released back to the atmosphere once exposed by retreating ice during deglaciation^1^. More recent work has highlighted the capacity of glacier surfaces to act as sinks for carbon-containing aerosols from anthropogenic or natural sources^2,3^. However, both these mechanisms represent passive processes of carbon storage and release by glaciers. Only in the last 15 years have glacial systems started to be considered as active cyclers of carbon, arising from the discovery that they include a range of aquatic environments^4^ which host abundant and diverse populations of microorganisms^5^ and are hot spots for biogeochemical weathering^6^. These processes create the potential for ice sheets to directly or indirectly impact the global carbon cycle (Fig. 1). Direct impacts include the release of greenhouse gases (carbon dioxide, CO_2_ and methane, CH_4_) during the microbial respiration of organic matter (OM) stored within ice sheets. Examples of indirect impacts include the fertilisation of downstream ecosystems, promoted by either the release of nutrient-rich glacial meltwaters^7–9^ or by subglacial meltwater-induced upwelling of nutrient replete marine water at tidewater glacier margins^10–14^. Ocean fertilisation by glaciers may be accompanied by significant CO_2_ drawdown by phytoplankton, intensifying the biological pump^15,16^.

The size of the carbon stores entombed in ice sheets^1,17,18^ and magnitude of nutrient supply to surface ocean waters by meltwaters and icebergs^8,19^ make it crucial to evaluate the role of ice sheets as open systems in regulating the global carbon cycle via the direct and indirect mechanisms identified in Fig. 1. Here, we draw upon advances over the last two decades to address the hypothesis that ice sheets have important direct and indirect impacts on the global carbon cycle (Fig. 1) at the present day, which are accentuated during periods of enhanced melt or ice discharge. In this Review, we first identify the key biogeochemical processes in ice sheets, and then go on to highlight in further discussions important indirect and direct impacts. We conclude this synthesis by evaluating the suggestion that ice sheets play a role in regulating the global carbon cycle with a novel analysis of the geological record over the last glacial-interglacial transition.

## A paradigm shift in ice sheet biogeochemical cycling

In 1999, the first communities of microorganisms were discovered beneath a valley glacier in the Swiss Alps^20^ driving a major shift in our world-view of glaciers and ice sheets as abiotic systems to extensive icy biomes^5^. Microbial communities have since been found ubiquitously in glacial systems^5^, ranging from sub-micron diameter liquid ice veins between ice crystals^21^ to large subglacial water bodies, such as Subglacial Lake Whillans situated 800 m beneath the West Antarctic Ice Sheet^22^. The survival of these seemingly diverse communities is due to their adaptation to a unique combination of physical and geochemical conditions that are not encountered anywhere else on Earth.

First, liquid water is abundant in ice sheets, generated by the melting of surface ice and snow and by geothermal/frictional heating and pressure melting of ice at the glacier bed. Surface melt is often widespread on the Greenland Ice Sheet and supplies melt to well-developed drainage systems at the ice sheet bed (where ice is at the pressure melting point)^23^. These drainage systems evolve seasonally from slow-inefficient distributed drainage to fast-efficient drainage (e.g., channels), with residence times shifting from months to <1 day^23^. In contrast, surface melt is largely absent in Antarctica, apart from in marginal locations^24,25^. Nonetheless, the bed of the Antarctic Ice Sheet is hydrologically active, with subglacial lakes, swamps, channels and groundwater aquifers fed by the melting of basal ice layers^4^. Water flowing in such long residence time drainage systems here may take years to decades to emerge at the ice margin^26^.

Second, the sliding of glaciers over their bedrock generates very fine, highly reactive rock flour by glacial crushing and grinding. This is the source of a potent mixture of electron acceptors (e.g., oxidised forms of sulphur and iron such as sulphate and Fe(III)), electron donors (e.g., OM, hydrogen^27^, as well as reduced elemental species such as Fe(II) in iron sulphide minerals), micro-nutrients and macro-nutrients, liberated by chemical dissolution. In the deep, dark and cold ecosystems beneath ice sheets, these rock-sourced redox pairs and nutrients promote the metabolism of a diverse mix of chemolithotrophic and chemoorganotrophic microorganisms, able to fix and supply autochthonous carbon to the wider subglacial ecosystem. In Antarctic Subglacial Lake Whillans, a large proportion of phylotypes in sediments were related to chemolithoautotrophic species that use reduced forms of sulphur, nitrogen and iron as energy sources^22^.

Third, as glaciers and ice sheets expand, they bury OM associated with soil, vegetation, lake and marine sediments. Furthermore, OM is deposited by aeolian processes onto the ice surface, originating from distant sources or from proximal surfaces surrounding the ice. This allochthonous material is supplemented by organic carbon fixed in situ by abundant autotrophic microorganisms in melting zones of ice sheet surfaces^22^, and thus, influences rates of ice melting by surface darkening (see Box 1)^28^.

Diverse oxidising and reducing conditions evolve in response to patterns of water flow beneath ice sheets, from fast flowing oxic channels to slower drainage through anoxic or hypoxic sediments and subglacial lakes. These hydrogeochemical environments create niches for microbial colonisation and permit a diverse range of biogeochemical processes to be supported across the redox spectrum, including sulphide oxidation^29^, denitrification^30,31^, sulphate^32^, and iron reduction^33,34^ and methanogenesis^35,36^. Well-developed hydrological systems, together with the release of icebergs via calving, create the potential for OM and nutrient transport out of the ice sheet and into downstream aquatic ecosystems (e.g., lakes, rivers, oceans). The release of fresh, buoyant subglacial meltwaters often some 100 s of m below the ocean surface at the margins of marine-terminating outlet glaciers also stimulates the upwelling of marine water and transport of associated nutrients to the ocean surface^10–14^. It is this combination of in situ microbial activity, dynamic processes of meltwater export and glacial advance/retreat cycles that creates an active connection between ice sheets, the ocean and the atmosphere (Fig. 1) and which we explore in subsequent sections.

Box 1 Microbial activity on ice sheet surfaces and the bioalbedo effect The bioalbedo effect refers to the darkening effect of biological impurities on snow and ice^177^. The melt zones of ice sheets are host to diverse communities of microorganisms that thrive during the summer months across a range of aqueous ecosystems including surface lakes, wet bare ice, snow and cryoconite holes (small cylindrical water-filled depressions, created by the solar heating of surface aggregates of mineral and organic particles and microbial cells, known as cryoconite)^178^. Bare ice surfaces are dominated by ice algae^179^, which are commonly green algae on the Greenland Ice Sheet^179–181^. In cryoconite holes, the dominant active taxa are cyanobacteria and alpha-proteobacteria and/or beta-proteobacteria^182^. The activity of these microorganisms has recently drawn attention via the hypothesis that the biologically driven accumulation of OM accelerates ice melting, via its effect upon ice surface darkening^28,183^. Net bioaccumulation depends upon the balance of CO_2_ fixed from the atmosphere by phototrophs and CO_2_ returned to the atmosphere by respiration, and is influenced by a wide range of physical and biogeochemical processes. Recent work on the Greenland Ice Sheet suggests that carbon fixation exceeds carbon consumption, making the ice sheet surface net autotrophic (i.e., a CO_2_ sink^113,183,184^) and enabling the accumulation of OM and pigmented algae^179^. Autochthonous OM is supplemented by wind-blown material, which becomes biologically altered on the ice sheet surface by the release of exopolymeric substances which bind particles^185^. The direct impact of the bioalbedo effect has yet to be fully quantified. However, initial investigations indicate that algal abundance on Greenland ice surfaces explains 70% of the variation ice reflectance, suggesting a significant impact of ice algae on ice surface albedo^28^. The resultant early exposure of underlying dark (low albedo) glacial ice following snowmelt has the potential to further enhance glacier melting^186^.

## Ice sheets as nutrient factories

A unique combination of intense physical erosion, active biogeochemical cycling and high meltwater fluxes from glacier systems, as described in the previous section, point towards the importance of ice sheets as direct or indirect sources and processors of a wide range of life-essential elements. There are three potential impacts of this glacially exported nutrient within the marine environment. First, direct fertilisation by the glacially exported nutrients themselves, including inorganic forms of N (e.g., NH_4_^+^, NO_3_^−^, NO_2_^−^) and P (PO_4_^3−^), organic forms of N and P, Si and micronutrients such as Fe (Box 2, Supplementary Tables 1 and 2)^30^. Second, indirect fertilisation by buoyant meltwater plumes entraining nutrient-rich marine waters as they rise up from depth^11,13,14,37,38^. Finally, indirect impacts via benthic recycling and liberation of nutrients from glaci-marine sediments^39^, which will in turn imprint upon upwelling oceanic waters. The release of nutrients to the surface ocean via these mechanisms has the potential to fertilise marine waters, stimulate changes in the plankton community composition^40^ and enhance primary production, export production and CO_2_ drawdown via the biological pump–an indirect impact on the carbon cycle. Deep ocean water and subglacial meltwaters tend to be enriched in different nutrient species, for example, subglacial meltwaters are enriched in crustal species such as Si and Fe but depleted in nitrate and phosphate, while deep marine waters are often enriched in nitrate and phosphate. Thus, the impacts of these two different mechanisms of nutrient release will be shaped by which nutrient they supply relative to that which is most limiting to phytoplankton.

By far the largest source of nutrients released directly via ice sheet meltwaters is the subglacial environment^8^, derived from bedrock by physical and biogeochemical weathering or from sequestered OM by biological activity^41^ (Box 2). Long flow paths (10 s to 1000 of km) result in enhanced meltwater residence times beneath ice sheets, which together with seasonally high meltwater pH (often > 8.5^42^), promote the chemical dissolution and alteration of nutrient-rich minerals such as aluminosilicates and P-bearing apatite. Thus, meltwaters and icebergs exported from ice sheets are also often enriched in bioavailable forms of dissolved and particulate phosphorous and silicon^42–44^. For example, yields of soluble reactive P generated in large ice sheet catchments are among the highest in the literature (17–27 kg P m^−2^ a^−1^)^42^, and an order of magnitude higher than from small valley glaciers^45,46^. Total P fluxes are an order of magnitude higher than those for other world rivers, largely sourced from minerals such as fluorapatite^42^. This suggests that high physical and chemical erosion rates in ice sheets^47^ are important in enhancing total P fluxes. Traditional notions of depressed silicate mineral weathering beneath small valley glaciers, inferred from low concentrations of dissolved Si in glacial runoff^48^, may also not hold for large ice sheets^43^. Recent work has shown significant concentrations of amorphous silicon (ASi) associated with suspended particulate matter in Greenland Ice Sheet runoff (~1% by mass). When this particulate component is included in total Greenland Ice Sheet Si fluxes via bulk runoff, the total flux rises by an order of magnitude to 50% of the total Arctic riverine Si flux^43^. Potential formation mechanisms include either production of ASi during rock grinding, a chemically leached surface layer or a precipitated weathering crust arising from dissolution/re-precipitation on freshly ground and leached particles^43^. Inclusion of ASi in total glacial Si fluxes has the potential to increase Si fluxes by an order of magnitude^43^. Glacially sourced ASi is highly soluble in seawater and its export, together with DSi, has the potential to change the oceanic Si isotopic signature and inventory on glacial-interglacial timescales^49^ and stimulate the productivity of diatoms and other siliceous organisms^43,44,50^.

Perhaps one of the most well studied nutrients released from glaciers and ice sheets is iron (Fe). The liberation of bioavailable Fe from bedrock via biogeochemical weathering (see Box 2) is thought to be important in fertilising ocean basins where phytoplankton are limited by Fe availability (e.g., the Southern Ocean, NW Pacific Ocean)^51,52^. A suite of microbially -mediated processes including silicate dissolution, sulphide oxidation^53^ and dissimilatory iron reduction^33,34^ generate ice sheet runoff enriched in filterable (operationally defined here as Fe which passes through a 0.2 or 0.45 μm filter) and highly reactive particulate forms of iron^54–56,57^ that contain bioavailable Fe(II)^58,59^. Icebergs are particularly important Fe exporters, containing enhanced concentrations of bioavailable (ascorbic acid extractable) iron (oxy)hydroxides (e.g., ferrihydrite) in iceberg rafted debris (IRD)^19^. There are likely important interactions between iron and other chemical species which are critical to consider within the context of wider ice sheet nutrient cycles, for example, the sorption of soluble reactive P (SRP) onto highly reactive Fe (oxy)hydroxide nanoparticles or colloids^42^.

In contrast to P, Si and Fe, concentrations of dissolved inorganic nitrogen in ice and snowmelt are low (nM to low μM)^42,44^ compared to deep ocean water (Supplementary Tables 1 and 2). While further NO_3_^−^ and NH_4_^+^ may be acquired by meltwaters in the subglacial environment by microbial activity, concentrations of dissolved N in glacial runoff are still in the low micromolar range^7,31^. The low N content of most bedrock types^60^ also means that the concentrations of N associated with suspended particulate material in ice sheet runoff are often below detection limit of analytical methods.

Organic phases of key nutrients are important in some contexts within ice sheets, resulting from the cycling of allochthonous OM and production of autochthonous OM. The organic phases of particulate P and N are measurable in ice sheet runoff, but comprise a much smaller proportion of the total N and P fluxes compared with those from Arctic rivers^31,42^. In comparison, the dissolved organic components of N and P in ice sheet meltwaters can be significant, acquired via the activity of micro-organisms on the glacier surface and at the bed. For example, dissolved organic N (DON) comprised 50% of the total dissolved N load of Greenland Ice Sheet runoff^31^. Dissolved organic matter (DOM) may also act as an important stabiliser for key redox-sensitive or phase-sensitive trace elements such as iron. A direct relationship between dissolved organic carbon (DOC) and dissolved iron observed in sub-Antarctic meltwaters was attributed to the stabilisation of filterable Fe (<0.45 μm) by complexation with DOM^56^, although further investigation is needed to determine if DOM-Fe linkage is important in glacial meltwaters.

**Fig. 2:**
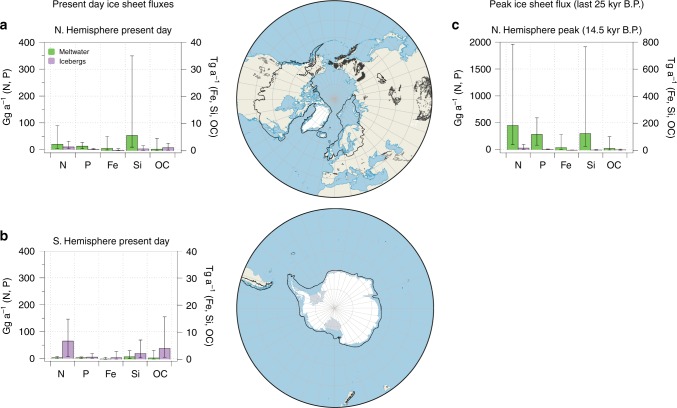
Fluxes of nutrients and organic carbon. Fluxes of bioavailable nutrients and organic carbon associated with subglacial meltwater and ice discharge from (**a**) northern and (**b**) southern hemisphere ice sheets at the present day and (**c**) northern hemisphere ice sheets during Meltwater Pulse 1a (14.5 kyr B.P.). LGM extents of ice sheets in both hemispheres are indicated by a black line in the maps. Nutrient fluxes and their uncertainty refer to the following species: N = DIN + loosely sediment bound NH_4_^+^ (also including loosely bound NO_3_^−^ for icebergs), *P* = DIP + bioavailable sediment bound P (MgCl_2_-P + NaOH-P), Fe = DFe (<0.02 μm) + CNFe (colloidal/nanoparticulate Fe, 0.02–0.45 μm) + SSFe (sediment-bound ascorbic acid extractable nanoparticulate Fe, e.g., ferrihydrite), Si = DSi + ASi and OC = POC + DOC. All fluxes include minimum, maximum and mid-range estimates calculated from published estimates of the range of nutrient concentrations and freshwater fluxes. Please see Supplementary Methods for flux calculations, error bars and the full dataset (note the different *y* axis scales in Northern Hemisphere peak fluxes)

Drawing upon previously published work we have generated the first global assessment of potential nutrient fluxes released directly from both ice sheets at the present day, also with indicative peak fluxes from northern hemisphere ice sheets during past periods of high melt rates, in this case, during Meltwater Pulse 1a at 14.5 kyr B.P. (see Supplementary Methods) (Fig. 2). The pattern that is immediately apparent from Fig. 2 is the important role of meltwater export in the northern hemisphere and of iceberg discharge in the south in delivering nutrients to the ocean–a reflection of the dominant water flux terms. Icebergs account for >95% of freshwater discharge from the Antarctic Ice Sheet and subglacial discharge dominates freshwater fluxes in the northern hemisphere, especially if most Greenland icebergs do not escape the extensive fjord systems^61^. High contemporary fluxes of bioavailable Fe and Si from ice sheets are observed (1.8 Tg a^−1^ Fe and 9.4 Tg a^−1^ Si), where >60% of these estimated Si and Fe fluxes are sourced from the Greenland Ice Sheet (Fig. 2, Supplementary Table 3). Present-day fluxes of Fe from the Greenland Ice Sheet are on a par with those associated with atmospheric dust to the North Atlantic^54^ and Fe associated with Antarctic icebergs exceeds fluxes via aeolian dust to the Southern Ocean by three orders of magnitude^61^. Particulate material is the dominant flux term (>95% of Si and Fe) for these nutrient species^8,62^ (Fig. 2). Bioavailable P and N fluxes from ice sheets are generally an order of magnitude (Gg a^−1^) lower than those for Fe, Si and organic carbon (OC, Tg a^−1^). However, these estimates exclude the more refractory mineral forms of P associated with particles (e.g., fluorapatite), which display fluxes orders of magnitude higher than the traditionally ascribed bioavailable P flux (taken to include soluble reactive and loosely bound P and P associated with Al-Fe minerals P)^42^. Fluxes of bioavailable P from ice sheets at the present day are of a similar order of magnitude (25 Gg a^−1^) to other riverine sources such as Arctic rivers (70 Gg a^−1^ bioavailable P)^42^. Present day N fluxes from ice sheets are moderate (100 Gg a^−1^ N, with 30 Gg a^−1^ from Greenland, 70 Gg a^−1^ from Antarctica) and exceed those from a major Arctic river (20 Gg a^−1^ for the Mackenzie River^63^) (Supplementary Table 3). Fluxes of nutrients associated with glacially driven upwelling of marine water at ice sheet margins are still poorly known. Studies of single fjords and marine terminating systems suggest that they could be highly significant for N and P “pumping”, and moderately significant for Si^10^. For example, in Sermilik and Illulisat Fjords (E Greenland), P fluxes via upwelling are 2–9 Gg N a^−1^ and those of N are 12–40 Gg P a^−1^, compared with estimates of  15 Gg P a^−1^ and  30 Gg N a^−1^ via meltwater and ice discharge from the entire Greenland Ice Sheet (Supplementary Table 3)^14^. For Si, upwelling fluxes are 14–66 Gg Si a^−1^ for these fjords, compared with 6300 Gg a^−1^ for the entire ice sheet (Supplementary Table 3)^14^.

Figure 2 indicates that nutrient fluxes from ice sheets were likely much higher during peak melt periods in the past, for example, during Meltwater Pulse 1a (MP1a at 14.5 ky B.P), when freshwater fluxes were c. 20× higher for meltwater and 3× higher for icebergs in the northern hemisphere–reflecting high meltwater export during rapid disintegration of ice sheets over Northern Europe and America. Thus, the potential export of Si and Fe at this time was likely very high (126 Tg a^−1^ and 21 Tg a^−1^, respectively). This flux of Si is almost equivalent to the total global river discharge of Si during the same time interval, which is notable given that the primary source of Si in the oceans is riverine inputs^43^. The impact of these boosted nutrient inputs on primary productivity in oceans bordering ice sheets during the last glaciation and glacial termination has been hinted at refs. ^52,64,65^, but is poorly constrained and requires a comprehensive modelling study in its own right. What is known about the fate of glacially exported nutrients at the present day is examined in the following section.

Box 2 Sources and cycling of nutrients **Atmospheric-derived nutrients**

The annual deposition of snow and subsequent transformation to ice locks up atmospheric-derived nutrients, such as nitrate^187^. Liberation of this ice-entombed nutrient is controlled by ice sheet hydrology and differs between ice sheets. On the Greenland Ice Sheet, atmospheric nutrients in snow and ice are released to surface meltwater streams in summer melt in the ablation zone^31,187^. In zones of ice sheets where surface air temperatures are well below 0 °C (e.g., most of the Antarctic Ice Sheet), zero surface melting means that this atmospheric nutrient release does not occur until the ice is advected to melting zones of the ice sheet bed. An exception is found in marginal regions, such as the McMurdo Dry Valleys, where solar radiation-driven melting of debris entombed in surface glacier ice causes localised melting and meltwater release to streams^24^.

**Ice sheet surfaces**

A wide range of ecosystems, such as cryoconite holes (see photo) on melting ice sheet surfaces, harbour productive microbiota with active nutrient cycling, including nitrogen fixation and nitrification^7,188–190^. These microbial processes further enhance the nutrient content of meltwaters prior to their discharge either to the ice sheet bed via moulins or at the margin. While nitrate concentrations can be several μM, surface meltwaters generally have much lower concentrations of crustally derived species such as P, Si and Fe^8^.

**Ice sheet beds**

Physical processes are vital in promoting cycling of nutrients at ice sheet beds via: (1) production of fresh mineral surfaces by glacial grinding; and (2) OM cycling, including that of ancient overridden OM. The immense capacity of glaciers to erode their underlying bedrocks via a combination of plucking and abrasion, generates an abundant supply of very fine rock flour. This gives glacial meltwater rivers a distinctive milky appearance, visible as turbid plumes when they enter downstream fjord and coastal waters^191^. Such flour is highly geochemically reactive, due to its large surface area: volume ratio and the exposure of reactive minerals such as iron sulphides and carbonates^48^. Traditionally, chemical weathering beneath ice sheets was assumed to be low to negligible^192^, but contemporary field studies in glacierised catchments indicate chemical weathering rates that exceed or are similar to the global mean^48^. Marine-based work using paleo-proxies also indicates that sub-ice sheet chemical dissolution of rock material is enhanced during deglaciation (21–7 kyr B.P) when large expanses of glacial sediments become exposed as the ice retreats^193,194^. The chemical weathering of glacial sediments is an important process driving the release of crustal nutrients, such as Si, P, Fe and other micronutrients in dissolved or bioavailable particulate forms. The first reaction to occur upon wetting of glacial flour is carbonate hydrolysis (e.g. hydrolysis of calcite, Eq. 1).

1 $${\rm{Ca}}{\rm{CO}}_{3}\left( {\rm{s}} \right) + {\rm{H}}_2{\rm{O}}\left( {\rm{l}} \right) \Leftrightarrow{\rm{Ca}}^{2 + }\left( {{\rm{aq}}} \right) + {\rm{HCO}}_3^ - \left( {{\rm{aq}}} \right) + {\rm{OH}}^ - \left( {{\rm{aq}}} \right)$$

Further chemical dissolution, including that of silicate minerals, is fuelled by carbonation reactions (including via microbial CO_2_ in some cases^6,195^), by protons released during the (microbial) oxidation of sulphide minerals (e.g. coupled calcite dissolution/sulphide oxidation via oxic or anoxic mechanisms, Eqs. 2 and 3 respectively)^29,53^ or via other forms of microbial weathering (e.g., release of metabolites such as organic acids).

2 $$16{\rm{CaCO}}_3\left( {\rm{s}} \right) + 4{\rm{FeS}}_2\left( {\rm{s}} \right) + 15{\rm{O}}_2\left( {{\rm{aq}}} \right) + 14{\rm{H}}_{\rm{2}}{\rm{O}}\left( {\rm{l}} \right) \Leftrightarrow 16{\rm{Ca}}^{2 + }\left( {{\rm{aq}}} \right) + 16{\rm{HCO}}_3^ - \left( {{\rm{aq}}} \right) + 8{\rm{SO}}_4^{2 - }\left( {{\rm{aq}}} \right) + 4{\rm{Fe}}\left( {{\rm{OH}}} \right)_3\left( {\rm{s}} \right)_{\left( {{\rm{ferric}}\;{\rm{oxyhydroxides}}} \right)}$$

3 $$16{\rm{CaCO}}_3\left( {\rm{s}} \right) + {\rm{FeS}}_2\left( {\rm{s}} \right) + 14{\rm{Fe}}^{3 + }\left( {{\rm{aq}}} \right) + 8{\rm{H}}_{\rm{2}}{\rm{O}}\left( {\rm{l}} \right) + \Leftrightarrow 16{\rm{Ca}}^{2 + }\left( {{\rm{aq}}} \right) + 16{\rm{HCO}}_3^ - \left( {{\rm{aq}}} \right) + 15{\rm{Fe}}^{2 + }\left( {{\rm{aq}}} \right) + 2{\rm{SO}}_4^{2 - }\left( {{\rm{aq}}} \right)$$

Silicate mineral dissolution generates dissolved Si in meltwaters, an important nutrient required by siliceous organisms such as diatoms and sponges. This process is thought to be enhanced beneath ice sheets relative to small valley glaciers, since long flow paths and prolonged meltwater residence times lead to the exhaustion of carbonate minerals in sediments or attainment of saturation with respect to calcite in meltwaters^6,196^. Iron cycling is also important in glacial systems, via sulphide oxidation (oxic and anoxic)^53^ and dissimilatory iron reduction^33^ (Eqs. 2, 3 and 4, respectively).

4 $${\rm{Acetate}} + 8{\rm{Fe}}^{3 + }\left( {{\rm{aq}}} \right) + 4{\rm{H}}_2{\rm{O}}_{({\rm{l}})} \Leftrightarrow 2{\rm{HCO}}_3^ - \left( {{\rm{aq}}} \right) + 8{\rm{Fe}}^{2 + }\left( {{\rm{aq}}} \right) + 9{\rm{H}}^ + \left( {{\rm{aq}}} \right)$$

OM buried beneath ice sheets during their formation, supplemented by new carbon production via chemolithoautotrophy^197^, provides another important substrate for microbial nutrient cycling in wet-based zones of ice sheet beds. Ammonium is thought to be acquired by microbial mineralisation of OM, subsequently fuelling populations of nitrifiers^22,198^. Heterotrophic respiration of subglacial OM utilising the full spectrum of electron acceptors along the redox continuum has been reported, including denitrification^198^, iron reduction^33^ (Eq. 4), sulphate reduction^32^ and methanogenesis^35,36^.

## Fate of glacially cycled nutrients

The fate of glacially exported or cycled nutrients and their impact on marine ecosystems depends upon a complex interplay of physical and biological processes, including proglacial/lake/fjord/estuarine filter effects (e.g., burial of reactive phases, benthic recycling, desorption/adsorption from particulates^39^), advection by ocean currents^66^, phytoplankton utilisation pathways^67–69^ and spatial patterns of phytoplankton nutrient limitation^51^. Observations of heightened biological activity in marine waters surrounding glaciers has been noted as early as 1938, when brown zones (representing turbid melt plumes) in front of tidewater glaciers were noted to be particularly productive regions for biota^70,71^, with more recent research reinforcing this connection^10,72^.

There is now compelling evidence for direct glacial nutrient fertilisation of marine ecosystems around the Antarctic Ice Sheet. Here, iron associated with subglacial meltwaters and icebergs is thought to stimulate enhanced marine primary productivity in the iron-limited Southern Ocean. Some of the highest concentrations of dissolved Fe in the Southern Ocean have been reported adjacent to Pine Island Glacier in the Amundsen Sea^73^, and in the western Antarctic Peninsula^74^, both inferred to derive from a glacier source. Modelling studies show that Southern Ocean primary productivity may be enhanced by up to 40% and export production by up to 30% by subglacial iron inputs^15,75,76^. Satellite remote sensing studies also suggest that Antarctic meltwater may be an important source of iron to coastal polynyas^77^ and that icebergs enhance primary productivity in the open and coastal Southern Ocean^78,79^, confirming previous field observations of bioavailable iron in iceberg rafted sediments^61^.

In contrast, runoff from northern hemisphere ice sheets often enters fjords and complex estuarine environments, modulating total fluxes to the ocean via flocculation and scavenging^38,80–82^. Recent studies suggest that direct inputs of nutrients from Greenlandic glaciers have a relatively small impact in stimulating fjord and coastal productivity because they do not supply large fluxes of nutrients (e.g., N and P) which limit phytoplankton in these waters and turbid melt plumes supress productivity in the summer months^10^. Here, the most important influence of the ice sheet is heralded to be the pumping of N-replete and P-replete deep ocean water to the surface during the upwelling of buoyant subglacial meltwater at marine-terminating glacier margins^10,13,14,37,44^. This indirect supply of nutrients has been linked to spring and summer phytoplankton blooms, and supports fisheries in the coastal zone^10^. An exception may be the direct export of silicon (both dissolved and amorphous particulate phases) in glacial meltwaters^83^, which has been demonstrated to support diatom blooms in Greenlandic fjords^11^, corroborating previous studies suggesting high silica concentrations and high diatom abundance in glacially fed fjords and coastal waters^84^.

While the impacts of glacially exported nutrients upon fjord and coastal productivity around Greenland may be limited, there is mounting evidence that there is a net export of glacial nutrients from land to the open ocean. For example, the concentrations of filterable and particulate iron in the surface waters at the mouths of Greenland fjords and in near coastal regions downstream of glacial meltwater inputs can be orders of magnitude higher than that in the open ocean^38,81,85,86^. Radium isotopes measured on surface ocean waters off the coast of Greenland strongly suggest the rapid offshore transport of glacial particles into shelf waters and into the open ocean^83^. Taking these inferences a step further, remote sensing and numerical modelling have showed a strong correlation between the timing and spatial extent of summer phytoplankton blooms in the Labrador Sea and the arrival of Greenland meltwater and subsequent patterns of advection via ocean currents^16^. The inferred driver for these summer (August, September) blooms is the advection of ice sheet derived Fe, which must be transported some 500 km offshore^16^. These summer/autumn blooms account for 40% of the annual net primary production for the region, indicating a process of regional significance^16^.

Increasing ice sheet freshwater discharge and glacier retreat^87^ in the 21st century are predicted to be accompanied by rising nutrient and organic carbon export from ice sheets^8,9^. However, this increase in glacially sourced nutrient fluxes may not be a simple linear function of the freshwater flux. For example, seasonal records of nutrient fluxes from a large Greenland outlet glacier in extreme melt years demonstrated that a doubling of glacial runoff was accompanied by a similar increase in dissolved nutrient loading^8^, but sediment-bound fluxes declined due to the intricate (supply-limited) relationship of particulate loading and bulk meltwater discharge^8^. Decreased glacier size, and hence erosion rates, may in fact also reduce sediment-bound nutrient export to the ocean, as seen for P export from two Greenland catchments of contrasting size^42^. In addition, expansion of the proglacial zone in land-terminating systems may drive fundamental shifts in microbial community size and composition and additional nutrient cycling steps may occur in proglacial aquatic ecosystems (e.g., lakes) prior to nutrient export to the ocean^88,89^. In regions where marine ice retreats to land, changes in the mechanisms of freshwater (and nutrient) input to ocean waters may occur. Thus, there may be a shift from sub-surface to surface (turbid) meltwater flows^10^ at the land-ocean margin, and cessation of the entrainment of nutrient-replete ocean water by rising buoyant melt plumes from depth at marine margins. These changes are likely to lead to light limitation and reduced macronutrient (N and P) supply, respectively, in surface waters^90^. They may explain the high biological productivity and fish catches found in the vicinity of fjords headed by marine terminating glaciers in Greenland compared with fjords dominated by land terminating glacier inputs^10^.

The above discussion highlights the importance of the fate of inorganic nutrients, supplied directly or indirectly via ice sheet meltwaters. However, ice sheets also act as significant stores of OM, which is either fixed by autotrophic microbial activity in situ^22^ or imported to the glacier system via wind-blown material (e.g., aeolian dust)^3^ or from biomes buried by ice during periods of glacial advance^17,18^. The cycling of this OM within glaciers and ice sheets creates the potential for a series of more direct impacts on the global carbon cycle (Fig. 1), which are discussed in the next section.

## Ice sheets as dynamic carbon stores

**Fig. 3:**
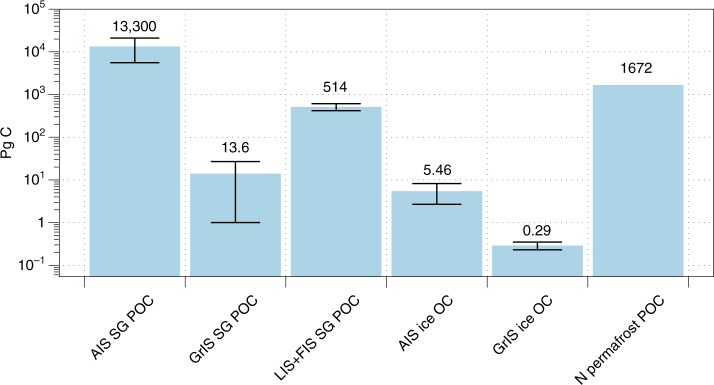
Carbon inventories for present day and former ice sheets. Data presented includes minimum, mid-range and maximum estimates. Antarctic Subglacial POC (AIS SG POC), minimum estimates are calculated assuming a sediment depth of 1 km, sedimentary basin areas of 1 × 10^6^ km^2^ for WAIS and 2.5 × 10^6^ km^2^ for EAIS, a rock density of 2650 kg m^-3^, a porosity of 0.3 and a POC of 0.1%^169^, maximum estimates are derived from ref. ^169^. and mid-range values as the average between the two, Greenland Ice Sheet Subglacial POC (GrIS SG POC, minimum estimates calculated assuming a POC of c. 0.2%^170^, sediment thicknesses of 0.1 m^171^ and a porosity of 0.4^172^, maximum estimates assuming a POC of 0.44%^36^, sediment thicknesses of 2 m^171^ and a porosity of 0.3^172^ and mid-range values as the average between the two), Laurentide + Fennoscandian Ice Sheet Subglacial POC (LIS + FIS SG POC, minimum, maximum and mid-range estimates from ref. ^18^), Antarctic Ice Sheet englacial POC + DOC (AIS ice OC, mid-range, minimum and maximum values from ref. ^9^, where the min/max values only include DOC due to lack of data), Greenland Ice Sheet ice-bound POC + DOC (GrIS ice OC, mid-range, minimum and maximum values from ref. ^9^, where the min/max values only include DOC due to lack of data) and Northern hemisphere permafrost (N permafrost POC)^97^

Pre-existing soil, vegetation, lake and marine sediments, and associated OM, are overridden and incorporated into sediments beneath ice sheets as they form^17,18^. Ice sheet surfaces may also act as extensive sinks for OM derived originally from terrestrial and anthropogenic sources^3,91–93^ or fixed via autotrophic biological activity^94^ (see Box 1). Some carbon reservoirs in ice sheets are thought to be vast. For example the estimated mass of particulate organic carbon (POC) held in sediments beneath the Antarctic Ice Sheet (6000–21,000 Pg C)^95^ is up to an order of magnitude greater than that associated with northern hemisphere permafrost^96^ and 1–2 orders of magnitude greater than estimates for the former Laurentide Ice Sheet (514 Pg C)^18^ (Fig. 3, methods in caption). This reflects the presence of extensive and thick subglacial sedimentary basins in Antarctica, thought to contain fossil OM originating from ancient marine sediments^97^. Smaller reserves of carbon are postulated to be present beneath the Greenland Ice Sheet (estimated at 0.5–27 Pg C, Fig. 3 for methods), where sediments are thinner but contain remains of ancient paleosols sequestered by the ice sheet during growth^36^. Carbon storage estimates (Fig. 3) have large error bounds, primarily due to uncertainties in sediment thicknesses and organic carbon content beneath ice sheets. However, they dwarf estimates of carbon pools held in englacial ice as dissolved organic carbon (DOC) or POC (Fig. 3) which are estimated at <10 Pg C for both present day ice sheets^9^. Evidence for the presence of sedimentary OM beneath ice sheets is found in the preservation of ancient paleosols in basal sediments and bulk meltwaters of the Greenland Ice Sheet^98,99^ and the presence of marine sediments and their geochemical influences beneath the West Antarctic Ice Sheet and in the McMurdo Dry Valleys^34,100^.

The fate of legacy OM beneath ice sheets is poorly known because of the inaccessibility of the subglacial environment. It is likely to include erosion and export of sedimentary OM (e.g., from soil, marine and lake sediments) from the ice sheet, storage in subglacial basins and frozen bed zones, and active respiration via microbial activity to CO_2_ or CH_4_. These greenhouse gases may be consumed by further microbial activity, transported in the dissolved/gas phases within a subglacial hydrological system or stored as gas hydrate in sediments (for CH_4_). Such processes of active carbon cycling create a direct link between ice sheets and carbon cycles of the atmosphere and oceans (Fig. 1) and are explored in the following sections.

### Erosion of sedimentary organic matter and fluxes downstream

Glacial erosion may lead to some OM removal or re-distribution over time beneath ice sheets. Indeed, where ice is warm at the bed and exhibits high rates of flow (e.g., close to the ice sheet margins), erosion rates could be considerable. For example, erosion rates of up to 0.6 mm a^−1^ have been plausibly modelled for Antarctic ice stream tributaries^101^ and rates of up to 5 mm a^−1^ are inferred at the margins of the Greenland Ice Sheet^47^. However, erosion rates in inland regions of ice sheets must be much smaller to be compatible with the preservation of sedimentary basins, such as those found in Antarctica even after 30 Ma of glaciation^102^. Consistent with this are calculated erosion rates in East Antarctica of 0.04 mm a^−1^ (Slessor Glacier^102^) and 0.001 mm a^−1^ (Lambert Basin^103^). The erosion and export of organic carbon from the polar ice sheets has recently become pertinent to the global carbon cycle, because of the potential for this material to become buried in long term geological carbon sinks^104^, such as the dark, oxygen-starved bottom waters of fjords^105^. The global rate of carbon burial in fjords is estimated to be 18 Tg a^−1^^105^^,^, but at many sites the proportions of old vs. newly produced OM are unknown. It is notable that the percentage organic carbon contents reported in Greenland fjord sediments (1.6%)^105^ are orders of magnitude higher than those recorded in suspended particulate material exported in runoff from large Greenland outlet glaciers (<0.1%)^106^.

The annual flux of DOC and POC in ice sheet meltwater and ice discharge is relatively small (present day total flux ~6 Tg C a^−1^, including ice-rafted debris (IRD)) compared to the total reserve held in ice or sediments (Fig. 3) and is equivalent to approximately 10% of the DOC and POC export from Arctic rivers^63,107^. Since erosion rates beneath ice sheets are generally low, material remaining under the ice is often old^36,99^. For example, if we assume a subglacial POC store in Greenland of 27 Pg C (Fig. 3), it would take some tens of thousands of years to export all of this organic carbon via runoff (assuming meltwater export of POC and DOC at <1 Tg a^−1^^106^^,^). This is consistent with the observation that the ^14^C radiocarbon ages of bulk POC associated with suspended particulate material in Greenland runoff are old (5–9 kyr) and become progressively older through a melt season as the snowline retreats inland^99^. We estimate that the amount of OC liberated via Antarctic subglacial meltwater discharge, is 0.17 Tg C a^−1^ as DOC and 0.33 Tg C a^−1^ as POC (using new data from Subglacial Lake Whillans for Antarctic DOC concentrations), which is comparable to previous estimates^9^ (see Supplementary Methods). DOC/POC release via Antarctic ice discharge is an order of magnitude higher at 4 Tg a^−1^.

Although fluxes of DOC and POC are relatively small on a global scale (<1% global land-ocean fluxes), a growing number of studies have observed that glacier derived DOC and POC are highly bioavailable^2,106^, and thus could be important in fuelling bacterial respiration in receiving aquatic ecosystems^2^. Observation of radiocarbon depleted (i.e., pre-aged) organic carbon on glacier surfaces and in glacial runoff also suggests that glacially exported DOC is also paradoxically ancient^3,92,93,108^. The source of this ancient DOC has been debated, as has the reason for its high bioavailability in comparison with non-glacial sources of DOC. Ice core data demonstrate a significant contribution of anthropogenic-derived carbon in glacier ice, concurrent with the onset of the industrial era^109,110^. The molecular composition of OM within Alaskan and Tibetan glaciers indicates high relative contributions of condensed aromatics, which is consistent with the presence of combustion derived OM (i.e., black carbon)^3,92,93^. Other studies have suggested an alternative explanation for aged DOC in glacial runoff (but not the glacier surface), arguing that it reflects cycling of overridden subglacial material (e.g., paleosols)^108^ or glacial erosion and associated reactivation of ancient OM in bedrock^111^. The high bioavailability of DOC emanating from glacial surfaces (and hence runoff) has been hypothesised to reflect OM sourced from the incomplete combustion of fossil fuels enriched in high abundances of nitrogen-rich aliphatic compounds^3^. However, more recent work also shows the potential role of microorganisms in cycling organic carbon to more bioavailable forms, giving rise to bioavailable DOC both on the glacier surface^112,113^ and at the glacier bed^114^. Future projections indicate that the total magnitude of DOC exported in glacier runoff between now and 2050 will be approximately 48 Tg C as annual glacier mass loss increases, particularly from mountain glaciers^9^. This increasing flux of glacier DOC provides a subsidy of bioavailable carbon to receiving streams, rivers, estuaries and coastal systems and will be disproportionally impactful in heavily glaciated coastal systems (e.g., Greenland, Gulf of Alaska, Patagonia) that are home to commercially important fisheries.

### Microbial conversion of organic matter to greenhouse gases

Sedimentary OM beneath ice sheets which is not eroded and exported from the glacier system is available as a substrate for subglacial microbial metabolism. An important fate for sedimentary OM buried beneath the ice is microbial respiration to greenhouse gases, and specifically CH_4_, under the anaerobic conditions that are inferred from a small number of data points recovered from ice sheet beds^22,115–117^. This may be supplemented by CH_4_ generated during erosion of underlying bedrock or from deep thermogenic sources in geothermically active zones^95^, such as West Antarctica^118^. Supersaturated CH_4_ concentrations of microbial origin have been reported recently in subglacial runoff draining from a small and much larger Greenlandic catchment^115,117^, Antarctic Subglacial Lake Whillans^116^ and in low temperature (1–10 °C) anaerobic incubation experiments of subglacial sediment sampled from a range of glaciers^36,95^. Concentrations of CH_4 _ > 15 times greater than atmospheric values have also been recorded in air expelled with meltwater via subglacial channels at the margins of a large glacier in SW Greenland^119^. However, there is high uncertainty regarding the magnitude of sinks for the CH_4_ produced, and how much CH_4_ is oxidised to CO_2_ before being released to the subglacial drainage system. This would reduce the positive radiative forcing associated with CH_4_ emissions from ice sheets by approximately a factor of 20. In very long residence time (i.e., years) subglacial hydrological systems, such as Subglacial Lake Whillans, results suggest that most CH_4_ generated in lake sediments is oxidised to CO_2_ before reaching the lake water^116^. However, observations from the Greenland Ice Sheet indicate that if subglacial meltwaters/sediments are transported rapidly via efficient channels to the margin during summer melt, this oxidative sink is much reduced and lateral fluxes of CH_4_ at the ice margin are high^117,119^ and rival those from other world rivers^117^. This suggests that subglacial hydrology is key in determining how much subglacial CH_4_ is released to the atmosphere_._ This uncertainty regarding CH_4_ export calls for wider study of CH_4_ production and export from large glacier catchments draining ice sheets.

Fluxes of CH_4_ in ice sheet runoff at the present day are poorly quantified. Conservative estimates via this manuscript suggest potential fluxes of <1 Tg a^−1^, employing modelled meltwater discharge from both ice sheets^120,121^ and concentrations of CH_4_ measured in runoff from two end member estimates for subglacial meltwaters (min concentrations = 0.024 μM^116^ and maximum concentrations of 83 μM^115^). However, high pressure/low temperature conditions that typify the beds of thick ice sheets should favour methane hydrate formation^17,95,122^, once saturation with respect to CH_4_ is attained in sediment porewaters^95^ (Box 3). Methane hydrate has yet to be directly detected beneath ice sheets, but numerical modelling suggests conditions conducive to its formation in Antarctic sedimentary basins and within the Greenland Ice Sheet interior, where ice and sediment thicknesses are >1 km^95,117^. There are numerous reports from marine sediments around the Antarctic Ice Sheet margin of high concentrations of CH_4_ in sediment porewaters^123–125^ and active and relict cold seeps have been found off the Antarctic Peninsula and South Georgia^126,127^. Recent drilling to marine sediments offshore from the Wilkes Land subglacial basin (via IODP cruise 318) showed extremely high concentrations of CH_4_ in sediment cores (up to 43,000 ppm)^128^. Such high concentrations were not found in cores further offshore. It is probable that these processes also prevail beneath the ice sheet, as indicated by the supersaturated CH_4_ concentrations in Subglacial Lake Whillans, West Antarctica^116^ (0.024 μM in lake waters to up to 300 µM in sediments).

**Fig. 4:**
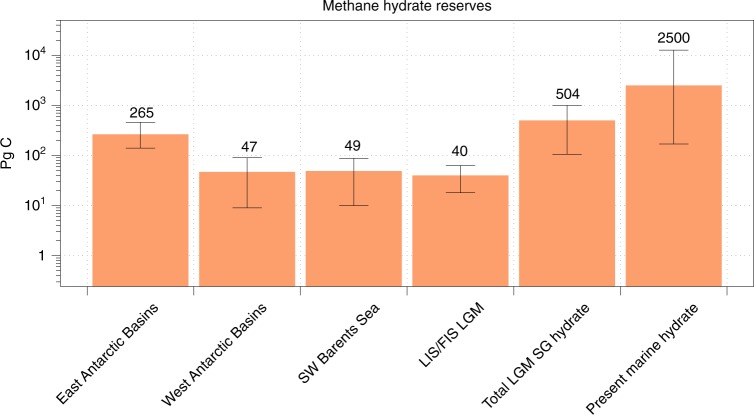
Potential methane hydrate reserves beneath present and former ice sheets. Data presented includes minimum, mid-range and maximum estimates of carbon reserves. East Antarctic Ice Sheet sedimentary basin methane hydrate + free gas (AIS hydrate, minimum and maximum values taken from ref. ^96^, with mid-range values as the average between the two), West Antarctic Ice Sheet sedimentary basin methane hydrate + free gas (WAIS hydrate, minimum and maximum estimates taken from ref. ^96^, with mid-range values as the average between the two), the SW Barents Sea LGM hydrate^165^ (mid-range values are the average of the minimum and maximum published values from ref. ^165^), Laurentide and Fennoscandian Ice Sheet estimates of potential methane hydrate (minimum and mid-range estimates from estimates of methane hydrate carbon modelled in ref. ^17^. and maximum estimates taken from calculations in ref. ^18^) (LIS/FIS LGM), Total LGM subglacial (SG) hydrate + free gas (sum of all data presented, with SW Barents Sea estimates scaled to the entire formerly glaciated Barents Sea Shelf^165^ hydrate scaled to the entire shelf), global present day marine methane hydrate, mid-range estimates (2500 Pg C) from ref. ^173^, minimum and maximum estimates from ref. ^174^ (170–12,700 Pg C)

There are substantial uncertainties regarding the magnitude of present day sub-ice sheet CH_4_ hydrate reserves because of the difficulties of accessing sediments in subglacial sedimentary basins. Global subglacial methane hydrate stocks at the present day are likely to be dominated by those in Antarctic sedimentary basins (estimated at up to 300 Pg C as methane hydrate and free gas^95^). At the LGM, the global sub-ice sheet hydrate reserve could have been much larger (>500 Pg C, 20% of the present day marine hydrate stocks), with hydrate also present beneath former northern hemisphere ice sheets^17,18,122^ (see Fig. 4 for details and calculation methods). The vulnerability of Antarctic subglacial CH_4_ hydrate reserves to destabilisation is high because of their predicted location around the continent’s periphery in sedimentary basins where ice thinning in a warming climate is probable. A priority for future ice sheet research is to establish the presence of methane hydrate beneath the Antarctic Ice Sheet, and to assess its vulnerability to destabilisation in a warming climate. Clues to this may be found in the paleo-record and are discussed below.

Box 3 Subglacial methane hydrate Methane hydrate (see inset image) is one of the three phases in which CH_4_ may be present, the other two being dissolved methane and free gas. Two critical conditions are required for methane hydrate to form in sediments. First, saturation with respect to methane must be attained in sediment porewaters. Second, pressure and temperature conditions must be suitable for hydrate formation—where low temperatures and high pressures favour hydrate over free gas for the saturated phase. These temperature and ice thickness pre-requisites are easily attained beneath thick ice sheets, and recent work shows in situ microbial methane production in subglacial sediments^35,36,115,116,168^. Methane hydrate itself comprises a methane molecule surrounded by a cage of water molecules, creating an ice-like structure. In marine sediments, methane hydrate may occupy up to a few percent of the sediment porosity. There are three additional factors that favour methane hydrate formation beneath ice sheets: a small and finite sulphate pool in subglacial sediments (via oxidation of sulphide minerals and/or overridden marine sediments) that reduces the potential for methane loss via Anaerobic Oxidation of Methane (AOM), frozen conditions creating a seal over the ice sheet bed, and finally glaciation periods of 10^5^–10^6^ years which promotes CH_4_ accumulation in sediment porewaters.

## Back to the future

Global glacier volumes are predicted to decrease over the 21st century^129^. The fastest present day relative net mass losses are apparent on mountain glaciers, but non-linear changes and high gross losses are expected for the Greenland and Antarctic ice sheets due to the collapse of floating ice tongues and marine ice sheet instability^129^. The potential impacts of ice sheet melting upon global sea level are well studied^129^, but the impacts of thinning ice and enhanced freshwater fluxes upon local, regional and global carbon cycles (Fig. 1) are poorly constrained. Disentangling the complex set of interactions between these future changes in ice sheet mass balance and the carbon cycle is a challenging task—measurement programmes typically cover short time periods (years) relative to changes in warming climate (decades) and there is a dearth of mechanistic models capable of simulating biogeochemical processes within ice sheets and their wider ramifications. In the final section of this paper, we draw upon the geological record to explore possible biogeochemical impacts of melting ice sheets in the past in order to provide clues to potential impacts in a future warming world. In doing so, we introduce potential indirect (ocean fertilisation) and direct (e.g., CH_4_ release from subglacial sedimentary basins) impacts of ice sheets on the global carbon cycle during past phases of ice sheet growth and retreat during the Quaternary (Fig. 1), which might form a strong focal point for future study.

### Fertilisation impacts of ice sheets

Climate warming over the last glacial-interglacial transition was accompanied by the disappearance of ice sheets over much of northern Europe and America^130^ and marginal retreat of the Antarctic Ice Sheet^131^. If indeed ice sheet freshwater export is a key source of nutrients for the world’s oceans, then the geological record should hint at shifts in the productivity of ocean basins bordering ice sheets synchronous with changes in ice sheet freshwater inputs (e.g., meltwater, icebergs). To introduce this idea and to provide some testable hypotheses for future work, we turn our attention to the Fe-limited^51^ Southern Ocean over the last glacial-interglacial transition. Here, the productivity of phytoplankton is limited primarily by the availability of Fe in surface ocean waters, and variations in the Fe supply over glacial-interglacial cycles has been argued as a plausible driver for CO_2_ drawdown^132^, via its influence upon the strength of the biological pump which controls carbon export to the deep ocean^133^. Specifically, enhanced Fe supply to the Sub-Antarctic zone of the Southern Ocean, and associated changes in export production, has been linked to the step decreases in atmospheric CO_2_ recorded in ice cores during the last glacial period, particularly during Marine Isotope Stages (MIS) 4/5 transition (c. 70 kyr B.P.) and around the LGM^134–137^.

There are a number of potential Fe sources in the Southern Ocean fertilisation game, all with their own spatial and temporal complexities. These include aeolian dust^132^, coastal sediments^138–141^, hydrothermal inputs^142^ and more recently, iceberg rafted debris (IRD)^143^ and meltwater from the Antarctic Ice Sheet^56,97,144^. Inferring the precise mechanism(s) driving Southern Ocean fertilisation at key periods of Earth’s history is challenging, in part due to the overlapping geographical impacts of different Fe sources. For example, inputs of Fe from dust, coastal sediments or icebergs are often concentrated in the Atlantic Sector of the Southern Ocean, via transport off Patagonia (dust^145^, including remobilised fine glacial sediments from Patagonian glaciers^58,146^) and via ocean currents along the so called iceberg alley adjacent to the Antarctic Peninsula. In general, discerning the importance of each of these potential Fe sources involves correlating past CO_2_ variations to reconstructions of iron/nutrient supply via elevated Fe inputs (the iron hypothesis^132^) and associated proxies for export production (e.g., opal accumulation)^147^.

**Fig. 5:**
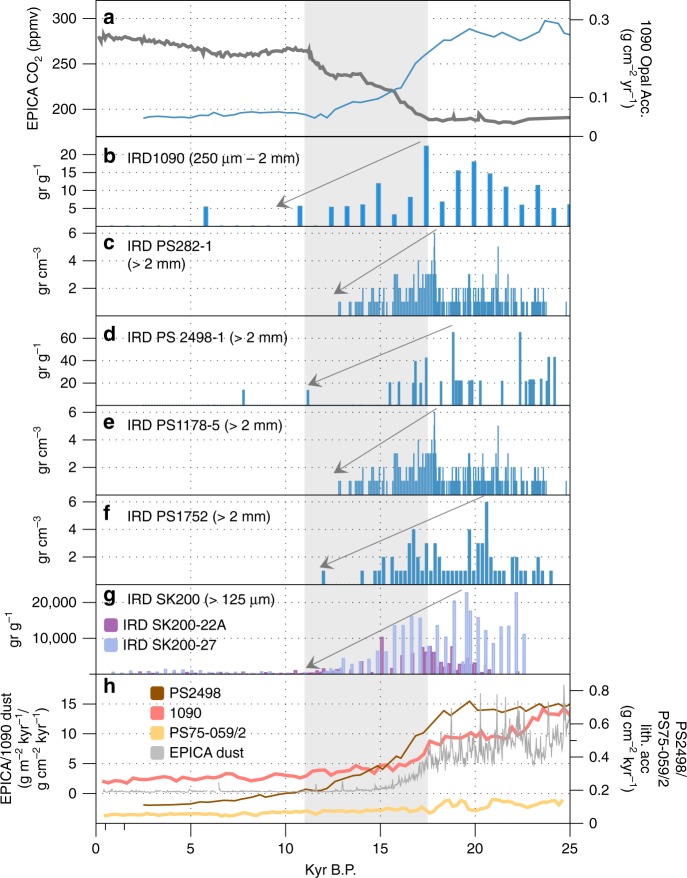
Temporal variability in the Southern Ocean. Temporal variation in atmospheric CO_2_, marine opal accumulation, Ice-rafted debris (IRD) and total lithogenic inputs to the Southern Ocean over the past 25 ka: **a** atmospheric CO_2_ concentrations (EPICA Dome C, dark grey line) and opal accumulation in sub-Antarctic cores 1090/PS2498-1174 (blue line)^151^, (**b**) IRD in core 1090^159^, (**c**) IRD in core PS282-1 where the IRD data are from ref. ^160^ and the Depth/Age model is from Ref. ^175^, (**d**) IRD in core PS2498-1 where the IRD data are from ref. ^160^ and the Depth/Age model is from ref. ^151^, (**e**) IRD core PS1778-5, where the IRD data are from ref. ^160^ and the Depth/Age model is from ref. ^176^, (**f**), IRD in core PS1752, where the IRD data are from ref. ^160^ and the Depth/Age model is from ref. ^177^. **g** IRD in cores SK200-22A and SK200-27 from the Indian Ocean sector of the Southern Ocean^161^. **h** Lithogenic fluxes to the Southern Ocean for marine cores 1090^137^, PS2498-1^151^ and PS75-059/2^152^ alongside the EPICA Dome C dust flux record. The shaded area indicates the period of maximum CO_2_ increase during deglaciation. See Supplementary Fig. 3 for core locations

Variation in aeolian dust supply to the Southern Ocean has attracted perhaps the greatest historical interest as an explanation for Fe fertilisation-driven changes in Southern Ocean productivity during the last glacial period, when enhanced inputs of wind-blown dust reflected changes in source area, as well as the strength and position of southern hemisphere westerlies^148^. Supporting the dust hypothesis for Southern Ocean fertilisation is the existence of excellent temporal records of past lithogenic fluxes (inferred to reflect dust inputs) to both the ice sheet^149^ and to marine sediments^136,150–152^, which lend themselves well to correlation with palaeo records of export production and atmospheric CO_2_^149^ (Fig. 5, Supplementary Fig. 2). For example, the EPICA Dome C ice core dust flux^149^ record highlights the last glacial period as a period of enhanced dust transport (14 mg m^−2^), and therefore, of potential Fe fertilisation of the Southern Ocean^149^. The fertilisation impacts of this Fe-bearing dust have been inferred in the SE Pacific and Atlantic (sub-Antarctic) sectors of the Southern Ocean at the LGM and have been linked to elevated rates of opal accumulation and overall export production^136,137,148,150,153,154^ (Fig. 5, Supplementary Fig. 2). They indicate Fe fertilisation of a northerly displaced opal belt–a zone of siliceous oozes and muds located between the Polar Front and the northern limit of seasonal sea ice which is associated with the upwelling of silica and nutrient-rich waters and relief of light limitation along the Antarctic Circumpolar Current (ACC) frontal system. In contrast, the Antarctic Zone of the Southern Ocean shows decreased opal export around the LGM^137,155^. A dusty source for core-bound lithogenic material in marine cores is implied by presence of terrestrial n-alkanes in the same cores, ascribed to the input of plant leaf waxes associated with terrestrial inputs of dust^156^.

A new player on the field of Southern Ocean Fe fertilisation is the Antarctic Ice Sheet. Early work on ice sheet contributions focussed upon iceberg rafted debris, which contains bioavailable Fe (oxy)hydroxide nanoparticles^61,86^ which are released from melting bergs as they drift often far offshore from the Antarctic continent^19,143^. Estimates of the potential bioavailable (ascorbic acid extractable) Fe fluxes associated with Antarctic IRD at the present day (Fig. 5, Supplementary Fig. 2) are c. 600 Gg Fe a^−1^ (range: 100– 2900Gg Fe a^−1^) (Fig. 2, Supplementary Table 3). These compare with Antarctic bioavailable Fe fluxes associated with dust which are several orders of magnitude lower at 0.56 Gg Fe a^−1^ for the present day^61^. More recently, ice shelf basal melt^73,157^, surface melt^56^, and subglacial meltwaters^15,74,144^ from the ice sheet have been proposed as potential sources of Fe to the Southern Ocean, but these melt (and Fe) inputs are likely limited to the coastal zone^15^.

Examination of records of IRD in several sub-Antarctic marine cores over the last glacial-interglacial transition^158–160^ (Fig. 5) suggest that Fe delivered by icebergs to the Southern Ocean could be an important segment of the deglacial Southern Ocean fertilisation, export production and CO_2_ story. In particular, elevated IRD deposition is recorded in sub-Antarctic cores between 22 kyr and 17 kyr B.P. when Antarctic ice volume was at its highest^161^ (Fig. 5). There is insufficient confidence in past iceberg flux estimates from the Antarctic Ice Sheet to calculate LGM fluxes of iceberg hosted Fe. However, we know from marine cores that IRD fluxes to the Atlantic sector of the sub-Antarctic Southern Ocean around the LGM were x10–20 present day fluxes, which is consistent with more intense IRD fertilisation of sub-Antarctic waters at this time (Fig. 5). Together, these findings suggest that a combination of high ice discharge and slower iceberg melting due to colder sea surface temperatures^158^ increased the supply of Fe-rich terrigenous material to sub-Antarctic waters during this interval, incidentally in a similar time frame to peak dust fluxes (Fig. 5). Later during deglaciation, we hypothesise that warmer ocean waters reduced iceberg transport offshore, such that Fe fertilisation of the sub-Antarctic zone substantially decreased and that of the Antarctic zone likely increased (Fig. 5).

It is difficult with the current data available to conclusively evaluate the role of iceberg-associated Fe in fertilising the Southern Ocean during the last glacial period alongside the more widely acclaimed dust. Certainly, the magnitude of present day IRD–Fe fluxes, when combined with the similarity in temporal and spatial patterns of IRD export and those for dust (Fig. 5), suggest that IRD may play a role in the Southern Ocean fertilisation story and warrants investigation. Unequivocally solving this mystery, however, requires a multi-pronged approach. First, it requires parallel study of a range of marine cores over a wide geographical area of the Southern Ocean to tease out the temporally variable contributions of both dust and IRD to the lithogenic flux record. Traditionally, IRD is assumed to account for the coarser fraction of lithogenic material in marine cores, which is variably defined (e.g., 250 μm−2 mm^158^, >1 mm^162^, >125 μm^160^). However, research on the grain size distribution of debris of glacial sediments, including those entombed in icebergs, often have an important fine (i.e., silt/clay) fraction^61,163^. Thus, while the presence of larger particles (e.g., sand) in marine cores might point towards IRD rather than dust inputs, the presence of silt/clays could reflect inputs from either IRD or dust. Geochemical provenance studies may help elucidate the precise origin of the lithogenic fraction in marine cores^150^, as may the use of biomarkers. The presence of n-alkanes derived from leaf waxes in the lithogenic material in marine cores is often interpreted as indicative of a dusty source^156^. While it seems unlikely that leaf waxes are present in sediments beneath the Antarctic Ice Sheet, the presence of these biomarkers in Antarctic IRD has not been evaluated. Finally, biogeochemical models have strong potential to reveal the magnitude of fertilisation impacts that could be possible in a glacial ocean via various Fe inputs, including dust and IRD. Model simulation of dust and ice sheet-sourced Fe impacts on Southern Ocean fertilisation and thus, productivity, have been attempted for a modern ocean and suggest strong influences by ice sheet Fe inputs^15,75,76^. However, these simulations have not been conducted for an LGM ocean. Part of the challenge of conducting such model studies is the grave uncertainty regarding potential Fe fluxes from the ice sheet to the ocean, which need to be better constrained. In summary, determining the role of iceberg-borne Fe in fertilising the Southern Ocean is no simple task, but has the potential to reveal powerful insights regarding the relationship between Fe export from the ice sheet (via melt and icebergs) and export production in the Southern Ocean, which may become more pertinent in a future warming world.

### Hydrate stability

**Fig. 6:**
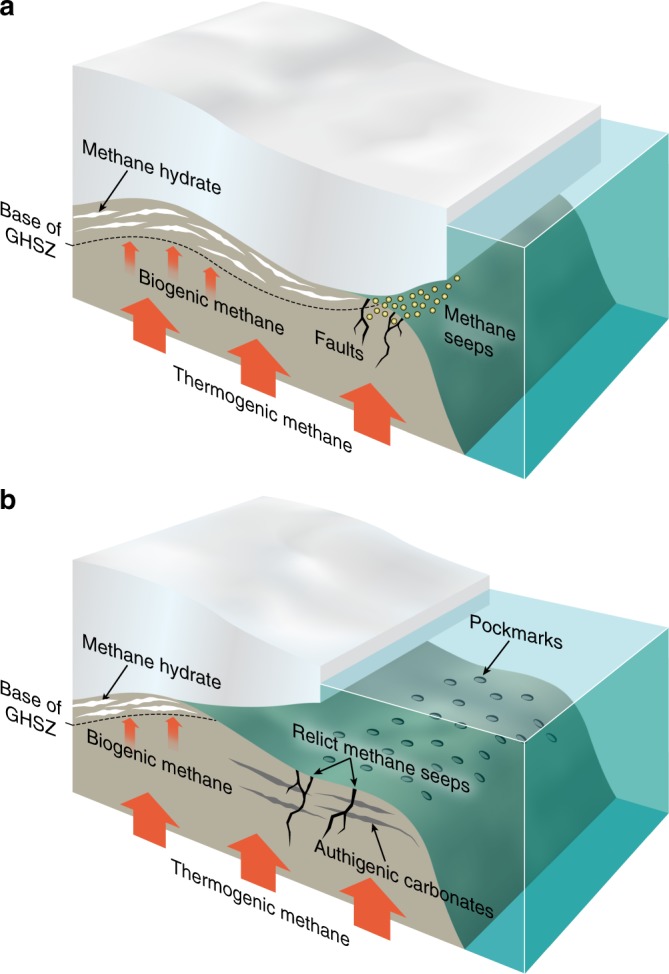
Model of ice sheet ice sheet impacts on hydrate reserves. A conceptual model illustrates the impact of ice sheet retreat and thinning on hydrate reserves beneath ice sheets, via their impact on the Gas Hydrate Stability Zone (GHSZ). (**a)** Conditions at peak glaciation, and (**b**) conditions after substantial ice sheet retreat. Following ice sheet retreat, relict features of hydrate destabilisation (e.g., authigenic carbonates, pock marks and relict cold seeps) may be evident on the formerly glaciated continental shelf

Turning our attention to potential direct effects of ice sheets upon the global carbon cycle, we examine the geological record for past periods of methane hydrate destabilisation beneath ice sheets, and associated CH_4_ release. Ice sheet thinning or retreat has the potential to dramatically alter in situ temperature and pressure conditions in sub-ice sheet sediments, triggering methane hydrate destabilisation and release of the resultant CH_4_ gas to the atmosphere (Fig. 6). Non-linear changes in Greenland and Antarctic Ice Sheet extent and thickness, due to marine ice sheet instability have the potential to trigger such a response, but are difficult to predict^129^. There is some evidence in marine sediments located close to the former margin of former Eurasian Ice Sheets that past phases of ice sheet retreat have been associated with methane hydrate destabilisation in subglacial sediments. Abundant pockmarks and authigenic carbonate crusts aligned to the position of the former LGM Scandinavian Ice Sheet margin are observed both in the Barents Sea and off the coast of Norway^122,164^. The former were interpreted to reflect fluid escape at the seafloor during (thermogenic) methane hydrate dissociation at depth, and the latter arise from AOM-driven saturation of sediment porewaters with respect to calcite. These features are contended to reflect CH_4_ release as the ice overburden was removed during the last deglaciation^122,164^ and provide compelling evidence that hydrate forms beneath ice sheets and is destabilised as the ice thins and retreats, in this case over a period of ~10 kyr^164^. Similar evidence has yet to be uncovered from the Antarctic Ice Sheet, where hydrate reservoirs are predicted at the present day^95^. Ice thickness reductions in marginal areas of the Antarctic Ice Sheet during the last deglaciation were significant (e.g., 300–800 m in East Antarctica^165^) and sufficient to destabilise potential subglacial hydrate reserves in these areas^95^. The potential impacts of such release events on atmospheric CH_4_ concentrations are unclear, and are complicated by the uncertainty regarding the fate of CH_4_ in marine waters (e.g., oxidation to the less potent greenhouse gas, CO_2_). For example, recent work off the coast of Svalbard has indicated that high CH_4_ gas fluxes from the seafloor instead stimulate enhanced marine productivity, likely due to indirect transportation of nutrient-rich water and CO_2_ (from dissociated CH_4_) from depth. In this case, the negative radiative forcing exceeded positive radiative forcing associated with CH_4_ release^166^. Resolving the influence of these opposing influences is important in determining the net impact of subglacial CH_4_ hydrate destabilisation on atmospheric CO_2_. If the CH_4_ is released to the atmosphere during non-linear ice retreat, it could impact the effectiveness of the UN Framework Convention on Climate Change emissions-based agreements to keep climate warming to a minimum of 2 °C by 2100.

### Challenges and next steps

**Fig. 7:**
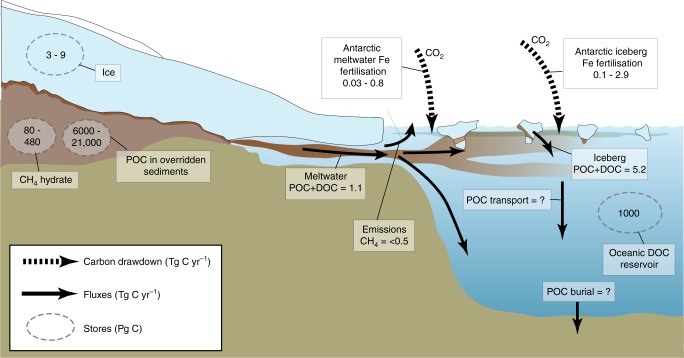
Stores and fluxes in present day ice sheets. A summary diagram indicating stores and fluxes of nutrients for present day ice sheets, and the predicted impact on CO_2_ (where data exists). The estimated size of carbon stores (Pg C) and Fluxes (Tg a^−1^)

The balance of evidence presented in the previous sections supports our opening hypothesis that ice sheets have an important impact on local, regional and global carbon cycles via a suite of direct and indirect effects. The estimated magnitude of these effects is summarised in Fig. 7. Sub-Antarctic sedimentary basins represent by far the largest single store of POC within ice sheets (6000–21,000 Pg C), and ice sheets may also contain significant reserves of climatically sensitive methane hydrate (500 Pg C at the LGM). Export terms (POC and DOC in runoff/icebergs) are comparatively small (<10 Tg a^−1^) but have potential regional importance for marine and lacustrine food webs due to the high lability of this material. The important indirect effect of meltwater and iceberg nutrient export upon the carbon cycle, via impacts on marine productivity, is illustrated by modelled 100 s Tg C a^−1^ of export production in the Southern Ocean associated with iron export from the ice sheet which reduces outgassing from the Southern Ocean by 30%^76^.

Scrutiny of the geological record also hints that these impacts were greater during periods of rapid ice sheet change over the last glacial-interglacial cycle. For example, a reduction in iceberg fluxes (and therefore, Fe) to the sub-Antarctic sector of the Southern Ocean from 20 to 11 kyr B.P. correlates in timing with the increase in atmospheric CO_2_ recorded in the EPICA Dome C ice core (Figure 5). On the other side of the world, the observation of pock marks aligned to the position of former ice sheets are consistent with the release of CH_4_ gas flares as the northern hemisphere deglaciated^122^. Despite these clues from the past, the impact of future climate warming in the Polar regions on the feedbacks between ice sheets and the global carbon cycle is highly uncertain. Predictions suggest that there may be increased fluxes of bioavailable nutrients to the ocean with rising freshwater discharge^8^ and destabilisation of hydrate reserves beneath ice sheets as ice thins^17,95^. These two processes have opposing potential impacts upon atmospheric CO_2_ concentrations, the former (nutrient fluxes to the ocean) acting as a negative feedback on warming and the latter (hydrate destabilisation) as a positive feedback. Discerning the relative importance of these impacts is hampered by a currently poor representation of ice sheet biogeochemical processes in global biogeochemical models due to gaps in data and understanding.

Thus, high uncertainty surrounds the estimates in Fig. 7, and future research must address several notable gaps in our current understanding, and hence predictive capabilities, of ice mass biogeochemical response to future warming. First, the basal regions of ice sheets are remote and challenging to access, and all predictions to date concerning the subglacial methane hydrate reserves or nutrient fertilisation potential, rely upon models calibrated to observations either in the laboratory or on smaller, more accessible valley glaciers. Direct access and sampling of deep subglacial aquatic environments such as subglacial lakes and sedimentary basins is essential, complemented by geophysical methods from which sub-ice conditions (e.g., hydrate) can be inferred. This requires a technological leap^167^. Second, a shift in focus towards the downstream impact of ice sheets will help drive new understanding. This should encompass research spanning the full land-to-ocean continuum, together with paleo-environmental studies that seek to detect past changes in the ocean and/or atmosphere systems in response to biogeochemical perturbations related to ice sheets. Large-scale uncertainty, however, still centres upon the fate of nutrients in fjords, beneath ice shelves and in nearshore coastal environments and in constraining changes to nutrient fluxes from glacial runoff, icebergs and via meltwater induced  upwelling as the freshwater flux increases. Last, coupled biogeochemical models, including feedbacks between the glacial cryosphere, atmosphere and oceans are required to test the sensitivity of carbon sinks or sources to changes in the terrestrial cryosphere. From a field where life was thought absent until two decades ago, the possibility for new discovery is immense, but demands creativity, tenacity and technological investment in order to narrow current uncertainties and to reveal the true role of ice sheets in the global carbon cycle.

## Supplementary information

Supplementary Information

## References

[1] Zeng N (2003). Glacial-interglacial atmospheric CO2 change—The glacial burial hypothesis. Adv. Atmos. Sci..

[2] Hood Eran, Fellman Jason, Spencer Robert G. M., Hernes Peter J., Edwards Rick, D’Amore David, Scott Durelle (2009). Glaciers as a source of ancient and labile organic matter to the marine environment. Nature.

[3] Stubbins Aron, Hood Eran, Raymond Peter A., Aiken George R., Sleighter Rachel L., Hernes Peter J., Butman David, Hatcher Patrick G., Striegl Robert G., Schuster Paul, Abdulla Hussain A. N., Vermilyea Andrew W., Scott Durelle T., Spencer Robert G. M. (2012). Anthropogenic aerosols as a source of ancient dissolved organic matter in glaciers. Nature Geoscience.

[4] Priscu, J. et al. Antarctic subglacial water:origin, evolution and ecology. In: *Polar Lakes and Rivers* (eds. Vincent, W. F., Laybourn-Parry, J.) (Oxford University Press, Oxford, United Kingdom 2009).

[5] Anesio AM, Laybourn-Parry J (2012). Glaciers and ice sheets as a biome. Trends Ecol. Evol..

[6] Wadham J. L., Tranter M., Skidmore M., Hodson A. J., Priscu J., Lyons W. B., Sharp M., Wynn P., Jackson M. (2010). Biogeochemical weathering under ice: Size matters. Global Biogeochemical Cycles.

[7] Hodson AJ, Mumford PN, Kohler J, Wynn PM (2005). The high arctic glacial ecosystem: new insights from nutrient budgets. Biogeochemistry.

[8] Hawkings JR (2015). The effect of warming climate on nutrient and solute export from the Greenland Ice Sheet. Geochem. Perspect. Lett..

[9] Hood E, Battin TJ, Fellman J, O’Neel S, Spencer RGM (2015). Storage and release of organic carbon from glaciers and ice sheets. Nat. Geosci..

[10] Meire Lorenz, Mortensen John, Meire Patrick, Juul-Pedersen Thomas, Sejr Mikael K., Rysgaard Søren, Nygaard Rasmus, Huybrechts Philippe, Meysman Filip J. R. (2017). Marine-terminating glaciers sustain high productivity in Greenland fjords. Global Change Biology.

[11] Meire Lorenz, Mortensen John, Rysgaard Søren, Bendtsen Jørgen, Boone Wieter, Meire Patrick, Meysman Filip J. R. (2016). Spring bloom dynamics in a subarctic fjord influenced by tidewater outlet glaciers (Godthåbsfjord, SW Greenland). Journal of Geophysical Research: Biogeosciences.

[12] Hopwood MJ (2018). Non-linear response of summertime marine productivity to increased meltwater discharge around Greenland. Nat. Commun..

[13] Kanna N (2018). Upwelling of macronutrients and dissolved inorganic carbon by a subglacial freshwater driven Plume in Bowdoin Fjord, Northwestern Greenland. J. Geophys. Res..

[14] Cape MR, Straneo F, Beaird N, Bundy RM, Charette MA (2019). Nutrient release to oceans from buoyancy-driven upwelling at Greenland tidewater glaciers. Nat. Geosci..

[15] Death R (2014). Antarctic ice sheet fertilises the Southern Ocean. Biogeosciences.

[16] Arrigo Kevin R., van Dijken Gert L., Castelao Renato M., Luo Hao, Rennermalm Åsa K., Tedesco Marco, Mote Thomas L., Oliver Hilde, Yager Patricia L. (2017). Melting glaciers stimulate large summer phytoplankton blooms in southwest Greenland waters. Geophysical Research Letters.

[17] Weitemeyer KA, Buffett BA (2006). Accumulation and release of methane from clathrates below the Laurentide and Cordilleran ice sheets. Glob. Planet. Change.

[18] Wadham JL, Tranter M, Tulaczyk S, Sharp M (2008). Subglacial methanogenesis: a potential climatic amplifier?. Glob. Biogeochem. Cycles.

[19] Raiswell R, Canfield DE (2012). The iron biogeochemical cycle past and present. Geochem. Perspect..

[20] Sharp M (1999). Widespread bacterial populations at glacier beds and their relationship to rock weathering and carbon cycling. Geology.

[21] Mader HM, Pettitt ME, Wadham JL, Wolff EW, Parkes RJ (2006). Subsurface ice as a microbial habitat. Geology.

[22] Christner BC (2014). A microbial ecosystem beneath the West Antarctic ice sheet. Nature.

[23] Chandler DM (2013). Evolution of the subglacial drainage system beneath the Greenland Ice Sheet revealed by tracers. Nat. Geosci..

[24] Gooseff MN, McKnight DM, Doran P, Fountain AG, Lyons WB (2011). Hydrological connectivity of the landscape of the McMurdo dry valleys, Antarctica. Geogr. Compass.

[25] Nicolas JP (2017). January 2016 extensive summer melt in West Antarctica favoured by strong El Niño. Nat. Commun..

[26] Fricker HA, Scambos T, Bindschadler R, Padman L (2007). An active subglacial water system in west Antarctica mapped from space. Science.

[27] Telling J (2015). Rock comminution as a source of hydrogen for subglacial ecosystems. Nat. Geosci..

[28] Stibal M (2017). Algae drive enhanced darkening of bare ice on the Greenland ice sheet. Geophys. Res. Lett..

[29] Bottrell SH, Tranter M (2002). Sulphide oxidation under partially anoxic conditions at the bed of the Haut Glacier d’Arolla, Switzerland. Hydrol. Process.

[30] Hodson A (2008). Glacial ecosystems. Ecol. Monogr..

[31] Wadham Jemma Louise, Hawkings Jonathan, Telling Jon, Chandler Dave, Alcock Jon, O&apos;Donnell Emily, Kaur Preeti, Bagshaw Elizabeth, Tranter Martyn, Tedstone Andre, Nienow Peter (2016). Sources, cycling and export of nitrogen on the Greenland Ice Sheet. Biogeosciences.

[32] Wadham JL, Bottrell S, Tranter M, Raiswell R (2004). Stable isotope evidence for microbial sulphate reduction at the bed of a polythermal high Arctic glacier. Earth Planet Sc. Lett..

[33] Nixon SL, Telling J, Wadham JL, Cockell CS (2016). Viable cold-tolerant iron-reducing microorganisms in geographically-isolated subglacial environments. Biogeosci. Discuss.

[34] Mikucki JA (2009). A contemporary microbially maintained subglacial ferrous “ocean”. Science.

[35] Boyd Eric S., Skidmore Mark, Mitchell Andrew C., Bakermans Corien, Peters John W. (2010). Methanogenesis in subglacial sediments. Environmental Microbiology Reports.

[36] Stibal M (2012). Methanogenic potential of Arctic and Antarctic subglacial environments with contrasting organic carbon sources. Glob. Change Biol..

[37] Juul-Pedersen T (2015). Seasonal and interannual phytoplankton production in a sub-Arctic tidewater outlet glacier fjord, SW Greenland. Mar. Ecol. Prog. Ser..

[38] Hopwood, M. J. et al. Seasonal changes in Fe along a glaciated Greenlandic fjord. *Front. Earth Sci.* **4**, 10.3389/feart.2016.00015 (2016).

[39] Wehrmann LM (2014). Iron and manganese speciation and cycling in glacially influenced high-latitude fjord sediments (West Spitsbergen, Svalbard): Evidence for a benthic recycling-transport mechanism. Geochim. Cosmochim. Acta.

[40] Slemmons KEH, Saros JE, Simon K (2013). The influence of glacial meltwater on alpine aquatic ecosystems: a review. Environ. Sci..

[41] Montross SN, Skidmore M, Tranter M, Kivimäki A-L, Parkes RJ (2013). A microbial driver of chemical weathering in glaciated systems. Geology.

[42] Hawkings J (2016). The Greenland Ice Sheet as a hot spot of phosphorus weathering and export in the Arctic. Glob. Biogeochem. Cycle.

[43] Hawkings, J. et al. Ice sheets as a missing source of silica to the world’s oceans. *Nat. Commun.* **8**, 10.1038/ncomms14198 (2017). 10.1038/ncomms14198 PMC5288494 28120824

[44] Meire L (2016). High export of dissolved silica from the Greenland Ice Sheet. Geophys. Res. Lett..

[45] Follmi KB, Hosein R, Arn K, Steinmann P (2009). Weathering and the mobility of phosphorus in the catchments and forefields of the Rhone and Oberaar glaciers, central Switzerland: implications for the global phosphorus cycle on glacial-interglacial timescales. Geochim. Cosmochim. Acta.

[46] Hodson A, Mumford P, Lister D (2004). Suspended sediment and phosphorus in proglacial rivers: bioavailability and potential impacts upon the P status of ice-marginal receiving waters. Hydrol. Process..

[47] Cowton T, Nienow P, Bartholomew I, Sole A, Mair D (2012). Rapid erosion beneath the Greenland ice sheet. Geology.

[48] Anderson SP (2007). Biogeochemistry of glacial landscape systems. Annu. Rev. Earth Planet. Sci..

[49] Hawkings, J. et al. The global silicon cycle impacted by meltwater runoff from past ice sheets. *Nat. Commun.* **9**, 10.1038/s41467-41018-05689-41461, (2018). 10.1038/s41467-018-05689-1 PMC6086862 30097566

[50] Burton, J. D. & Lis, P. S. Processes of supply and removal of dissolved silicon in the oceans. *Geochim. Cosmochim. Acta* **37**, 1761–1773 (1973).

[51] Moore CM (2013). Processes and patterns of oceanic nutrient limitation. Nat. Geosci..

[52] Müller J (2018). Cordilleran ice-sheet growth fueled primary productivity in the Gulf of Alaska, northeast Pacific Ocean. Geology.

[53] Harrold ZR (2016). Aerobic and anaerobic thiosulfate oxidation by a cold-adapted, subglacial chemoautotroph. Appl. Environ. Micro..

[54] Hawkings, J. et al. Ice sheets as a significant source of highly reactive nanoparticulate iron to the oceans. *Nat. Commun.* **5**, 10.1038/ncomms4929 (2014). 10.1038/ncomms4929 PMC4050262 24845560

[55] Bhatia MP (2013). Greenland meltwater as a significant and potentially bioavailable source of iron to the ocean (vol 6, pg 274, 2013). Nat. Geosci..

[56] Hodson, A. et al. Climatically sensitive transfer of iron to maritime Antarctic ecosystems by surface runoff. *Nat Commun.* **8**, 10.1038/ncomms14499, (2017). 10.1038/ncomms14499 PMC5316877 28198359

[57] Stevenson, E. I., Fantle, M. S., Das, S. B., Williams, H. M. & S. M. Aciego, S.M. The iron isotopic composition of subglacial streams draining the Greenland ice sheet. *Geochim. Cosmochim. Acta* **213**, 237–254 (2017).

[58] Shoenfelt EM, Winckler G, Lamy F, Anderson RF, Bostick BC (2018). Highly bioavailable dust-borne iron delivered to the Southern Ocean during glacial periods. Proc. Natl Acad. Sci..

[59] Hawkings JR (2018). Biolabile ferrous iron bearing nanoparticles in glacial sediments. Earth Planet Sci. Lett..

[60] Holloway JoAnn M., Dahlgren Randy A. (2002). Nitrogen in rock: Occurrences and biogeochemical implications. Global Biogeochemical Cycles.

[61] Raiswell, R. et al. Potentially bioavailable iron delivery by iceberg hosted sediments and atmospheric dust to the polar oceans. *Biogeosci. Discuss*10.5194/bg-2016-5120 (2016).

[62] Eiriksdottir ES, Gislason SR, Oelkers EH (2015). Direct evidence of the feedback between climate and nutrient, major, and trace element transport to the oceans. Geochim. Cosmochim. Acta.

[63] Holmes RM (2012). Seasonal and annual fluxes of nutrients and organic matter from large rivers to the arctic ocean and surrounding seas. Estuar. Coast.

[64] Gil Isabelle, M., Keigwin Lloyd, D., Abrantes Fatima, G. Deglacial diatom productivity and surface ocean properties over the Bermuda Rise, northeast Sargasso Sea. *Paleoceanography* **24**, 10.1029/2008PA001729 (2009).

[65] Hendy IL (2015). Ironing out carbon export to the deep ocean. Proc. Natl Acad. Sci..

[66] Hopwood MJ, Bacon S, Arendt K, Connelly DP, Statham PJ (2015). Glacial meltwater from Greenland is not likely to be an important source of Fe to the North Atlantic. Biogeochemistry.

[67] Shaked, Y. & Lis, H. Disassembling iron availability to phytoplankton. *Front. Microbiol.* **3**, 10.3389/fmicb.2012.00123 (2012). 10.3389/fmicb.2012.00123 PMC3328120 22529839

[68] Rubin M, Berman-Frank I, Shaked Y (2011). Dust- and mineral-iron utilization by the marine dinitrogen-fixer Trichodesmium. Nat. Geosci..

[69] Kuma K, Matsunaga K (1995). Availability of colloidal ferric oxides to coastal marine phytoplankton. Mar. Biol..

[70] Hartley AE, Dunbar G (1938). On the hydrographic mechanism of the so-called brown zones associated with tidal glaciers. J. Mar. Res.

[71] Greisman P (1979). On upwelling driven by the melt of ice shelves and tidewater glaciers. Deep Sea Res. Part A Oceanogr. Res. Pap..

[72] Lydersen C (2014). The importance of tidewater glaciers for marine mammals and seabirds in Svalbard, Norway. J. Mar. Syst..

[73] Gerringa LJA (2012). Iron from melting glaciers fuels the phytoplankton blooms in Amundsen Sea (Southern Ocean): iron biogeochemistry. Deep-Sea Res. Part II.

[74] Annett AL (2015). Comparative roles of upwelling and glacial iron sources in Ryder Bay, coastal western Antarctic Peninsula. Mar. Chem..

[75] Lancelot C (2009). Spatial distribution of the iron supply to phytoplankton in the Southern Ocean: a model study. Biogeosciences.

[76] Laufkötter C, Stern AA, John JG, Stock CA, Dunne JP (2018). Glacial iron sources stimulate the southern ocean carbon cycle. Geophys. Res. Lett..

[77] Arrigo KR, Van Dijken GL, Strong AL (2015). Environmental controls of marine productivity hot spots around Antarctica. J. Geophys. Res..

[78] Duprat LPAM, Bigg GR, Wilton DJ (2016). Enhanced Southern Ocean marine productivity due to fertilization by giant icebergs. Nat. Geosci..

[79] Smith KL (2007). Free-drifting icebergs: hot spots of chemical and biological enrichment in the Weddell Sea. Science.

[80] Markussen, T. N., Elberling, B., Winter, C., Andersen, T. J. Flocculated meltwater particles control Arctic land-sea fluxes of labile iron. *Sci. Rep.* **6**, 10.1038/srep24033 (2016). 10.1038/srep24033 PMC4822144 27050673

[81] Schroth AW, Crusius J, Hoyer J, Campbell R (2014). Estuarine removal of glacial iron and implications for iron fluxes to the ocean. Geophys. Res. Lett..

[82] Zhang R (2015). Transport and reaction of iron and iron stable isotopes in glacial meltwaters on Svalbard near Kongsfjorden: from rivers to estuary to ocean. Earth Planet Sci. Lett..

[83] Hendry KR (2019). The biogeochemical impact of glacial meltwater from Southwest Greenland. Prog. Oceano..

[84] Krawczyk D, Witkowski A, Waniek J, Wroniecki M, Harff J (2014). Description of diatoms from the Southwest to West Greenland coastal and open marine waters. Polar Biol..

[85] Schroth Andrew W., Crusius John, Chever Fanny, Bostick Benjamin C., Rouxel Olivier J. (2011). Glacial influence on the geochemistry of riverine iron fluxes to the Gulf of Alaska and effects of deglaciation. Geophysical Research Letters.

[86] Raiswell, R., Hawkings, J., Elsenousy, A., Death, R., Tranter, M. & Wadham, J. Iron in Glacial Systems: Speciation, Reactivity, Freezing Behavior, and Alteration During Transport. *Front. Earth Sci.* **6**, 222 (2018).

[87] Bamber Jonathan, van den Broeke Michiel, Ettema Janneke, Lenaerts Jan, Rignot Eric (2012). Recent large increases in freshwater fluxes from Greenland into the North Atlantic. Geophysical Research Letters.

[88] Bradley James A., Singarayer Joy S., Anesio Alexandre M. (2014). Microbial community dynamics in the forefield of glaciers. Proceedings of the Royal Society B: Biological Sciences.

[89] Sommaruga R. (2015). When glaciers and ice sheets melt: consequences for planktonic organisms. Journal of Plankton Research.

[90] Schloss IR (2014). On the phytoplankton bloom in coastal waters of southern King George Island (Antarctica) in January 2010: an exceptional feature?. Limnol. Oceano..

[91] Singer GA (2012). Biogeochemically diverse organic matter in Alpine glaciers and its downstream fate. Nat. Geosci..

[92] Spencer RGM (2014). Source and biolability of ancient dissolved organic matter in glacier and lake ecosystems on the Tibetan Plateau. Geochim. Cosmochim. Acta.

[93] Spencer Robert G M, Vermilyea Andrew, Fellman Jason, Raymond Peter, Stubbins Aron, Scott Durelle, Hood Eran (2014). Seasonal variability of organic matter composition in an Alaskan glacier outflow: insights into glacier carbon sources. Environmental Research Letters.

[94] Stibal M (2012). Environmental controls on microbial abundance and activity on the greenland ice sheet: a multivariate analysis approach. Micro. Ecol..

[95] Wadham JL (2012). Potential methane reservoirs beneath Antarctica. Nature.

[96] Tarnocai C., Canadell J. G., Schuur E. A. G., Kuhry P., Mazhitova G., Zimov S. (2009). Soil organic carbon pools in the northern circumpolar permafrost region. Global Biogeochemical Cycles.

[97] Wadham JL (2013). The potential role of the Antarctic Ice Sheet in global biogeochemical cycles. Earth Environ. Sci. Trans. R. Soc. Edinb..

[98] Souchez, R. et al. Gas isotopes in ice reveal a vegetated central Greenland during ice sheet invasion. *Geophys. Res. Lett*. **33**, 10.1029/2006gl028424 (2006).

[99] Kohler T. J., Žárský J. D., Yde J. C., Lamarche-Gagnon G., Hawkings J. R., Tedstone A. J., Wadham J. L., Box J. E., Beaton A. D., Stibal M. (2017). Carbon dating reveals a seasonal progression in the source of particulate organic carbon exported from the Greenland Ice Sheet. Geophysical Research Letters.

[100] Michaud, A. B. et al. Solute sources and geochemical processes in Subglacial Lake Whillans, West Antarctica. *Geology*10.1130/g37639.37631 (2016).

[101] Bougamont M, Tulaczyk S (2003). Glacial erosion beneath ice streams and ice-stream tributaries: constraints on temporal and spatial distribution of erosion from numerical simulations of a West Antarctic ice stream. Boreas.

[102] Bamber JL (2006). East Antarctic ice stream tributary underlain by major sedimentary basin. Geology.

[103] Jamieson SSR, Hulton NRJ, Sugden DE, Payne AJ, Taylor J (2005). Cenozoic landscape evolution of the Lambert basin, East Antarctica: the relative role of rivers and ice sheets. Glob. Planet. Change.

[104] Hilton RG (2015). Erosion of organic carbon in the Arctic as a geological carbon dioxide sink. Nature.

[105] Smith RW, Bianchi TS, Allison M, Savage C, Galy V (2015). High rates of organic carbon burial in fjord sediments globally. Nat. Geosci..

[106] Lawson E (2013). Greenland ice sheets exports labile organic carbon to the Arctic oceans. Biogeosciences Discuss..

[107] McClelland JW (2016). Particulate organic carbon and nitrogen export from major Arctic rivers. Global Biogeochem. Cycle.

[108] Bhatia MP (2013). Organic carbon export from the Greenland ice sheet. Geochim. Cosmochim. Acta.

[109] Grannas AM, Hockaday WC, Hatcher PG, Thompson LG, Mosely-Thompson E (2006). New revelations on the nature of organic matter in ice cores. J. Geophys. Res. Atmospheres.

[110] Jenk TM (2006). Radiocarbon analysis in an Alpine ice core: record of anthropogenic and biogenic contributions to carbonaceous aerosols in the past (1650–1940). Atmos. Chem. Phys..

[111] Tranter, M. Grand challenge for low temperature and pressure geochemistry—sparks in the dark, on Earth, Mars, and throughout the Galaxy. *Front. Earth Sci.* **3**, 10.3389/feart.2015.00069 (2015).

[112] Smith HJ (2016). Biofilms on glacial surfaces: hotspots for biological activity. Npj Biofilms Micro..

[113] Musilova M (2017). Microbially-driven export of organic carbon from the Greenland ice sheet. Nat. Geosci..

[114] O’Donnell EC (2016). Identification and analysis of low-molecular-weight dissolved organic carbon in subglacial basal ice ecosystems by ion chromatography. Biogeosciences.

[115] Dieser M (2014). Molecular and biogeochemical evidence for methane cycling beneath the western margin of the Greenland Ice Sheet. ISME J..

[116] Michaud AB (2017). Microbial oxidation as a methane sink beneath the West Antarctic Ice Sheet. Nat. Geosci..

[117] Lamarche-Gagnon G (2019). Greenland melt drives continuous export of methane from the ice-sheet bed. Nature.

[118] Maule CF, Purucker ME, Olsen N, Mosegaard K (2005). Heat flux anomalies in Antarctica revealed by satellite magnetic data. Science.

[119] Christiansen JR, Jørgensen CJ (2018). First observation of direct methane emission to the atmosphere from the subglacial domain of the Greenland Ice Sheet. Sci. Rep..

[120] Pattyn F (2010). Antarctic subglacial conditions inferred from a hybrid ice sheet/ice stream model. Earth Planet Sci. Lett..

[121] Bamber JL (2018). Land ice freshwater budget of the arctic and north atlantic oceans: 1. data, methods, and results. J. Geophys. Res..

[122] Portnov, A., Vadakkepuliyambatta, S., Mienert, J., Hubbard, A. Ice-sheet-driven methane storage and release in the Arctic. *Nat. Commun*. **7**, 10.1038/ncomms10314 (2016). 10.1038/ncomms10314 PMC4729839 26739497

[123] Claypool, G., Lorensen, T. D., Johnson, C. A. Authigenic carbonates, methane generation, and oxidation in continental rise and shelf sediments, ODP leg 188, Sites 1165 and 1166, Prydz Bay, Offshore Antarctica (Prydz Bay). In: *Proceedings of the Ocean Drilling Programme, Scientific Results* (Ocean Drilling Program, Texas A&M University, College Station, TX77845-9547, U.S.A. 2003).

[124] Barker, P. F., Camerlenghi, A., Acton, G. D. Leg 178 Summary. In: *Proceedings of the Ocean Drilling Programme, Initial Reports* (Ocean Drilling Program, Texas A&M University, College Station, TX77845-9547, U.S.A. 1999).

[125] Lonsdale MJ (1990). The relationship between silica diagensis, methane and seismic reflections on the South Orkney Microcontinent. Proc. Ocean Drill. Program. Sci. Results.

[126] Domack E (2005). A chemotrophic ecosystem found beneath Antarctic ice shelf. EOS Trans. Am. Geophys. Union.

[127] Römer M (2014). First evidence of widespread active methane seepage in the Southern Ocean, off the sub-Antarctic island of South Georgia. Earth Planet Sc. Lett..

[128] Scientists, E. Wilkes Land Glacial History: Cenozoic East Antarctic Ice Sheet evolution from Wilkes Land margin sediments. In: *Integrated Ocean Drilling Program* (Ocean Drilling Program, Texas A&M University, College Station, TX77845-9547, U.S.A 2010).

[129] IPCC. *Climate Change 2013-The Physical Science Basis* (2013).

[130] Dyke, A. S., Prest, V. K. Paleogeography of northern North America years ago. In: *Map/Geological Survey of Canada 1703A.* Geological Survey of Canada, Department of Enery, Mines, and Resources: Copies of this map may be obtained from the Geological Survey of Canada, (1987).

[131] Clark PU (2009). The last glacial maximum. Science.

[132] Martin PG (1999). Glacial-interglacial CO2 change: the iron hypothesis. Paleoceanography.

[133] Sabine CL (2004). The oceanic sink for anthropogenic CO2. Science.

[134] Hain Mathis P., Sigman Daniel M., Haug Gerald H. (2010). Carbon dioxide effects of Antarctic stratification, North Atlantic Intermediate Water formation, and subantarctic nutrient drawdown during the last ice age: Diagnosis and synthesis in a geochemical box model. Global Biogeochemical Cycles.

[135] Kohfeld KE, Chase Z (2017). Temporal evolution of mechanisms controlling ocean carbon uptake during the last glacial cycle. Earth Planet Sci. Lett..

[136] Martínez-García A (2014). Iron fertilization of the subantarctic ocean during the last ice age. Science.

[137] Jaccard SL (2013). Two modes of change in southern ocean productivity over the past million years. Science.

[138] Elrod Virginia A., Berelson William M., Coale Kenneth H., Johnson Kenneth S. (2004). The flux of iron from continental shelf sediments: A missing source for global budgets. Geophysical Research Letters.

[139] Blain S (2007). Effect of natural iron fertilization on carbon sequestration in the Southern Ocean. Nature.

[140] Pollard RT (2009). Southern Ocean deep-water carbon export enhanced by natural iron fertilization. Nature.

[141] Sherrell, R. M., Annett, A. L., Fitzsimmons, J. N., Roccanova, V. J. & Meredith, M. P. A ‘shallow bathtub ring’ of local sedimentary iron input maintains the Palmer Deep biological hotspot on the West Antarctic Peninsula shelf. *Philos. Trans. Royal Soc. A* **376**, 20170171 (2018). 10.1098/rsta.2017.0171 PMC5954470 29760114

[142] Tagliabue A (2010). Hydrothermal contribution to the oceanic dissolved iron inventory. Nat. Geosci..

[143] Raiswell, R., Benning, L. G., Tranter, M., Tulaczyk, S. Bioavailable iron in the Southern Ocean: the significance of the iceberg conveyor belt. *Geochem. Trans.* **9**, 10.1186/1467-4866-1189-1187 (2008). 10.1186/1467-4866-9-7 PMC2440735 18513396

[144] Monien D (2017). Meltwater as a source of potentially bioavailable iron to Antarctica waters. Antar. Sci..

[145] Petit J-R (1999). Climate history history of the past 420 000 years from the Vostok ice core, Antarctica. Nature.

[146] Sugden DE, McCulloch RD, Bory AJM, Hein AS (2009). Influence of Patagonian glaciers on Antarctic dust deposition during the last glacial period. Nat. Geosci..

[147] Broecker W (1982). Ocean chemistry during glacial time. Geochim Cosmochim. Acta.

[148] Kohfeld KE (2013). Southern hemisphere westerly wind changes during the last glacial maximum: paleo-data synthesis. Quat. Sci. Rev..

[149] Augustin, L. et al. Eight glacial cycles from an Antarctic ice core. *Nature* **429**, 623–628 (2004). 10.1038/nature02599 15190344

[150] Anderson Robert F., Barker Stephen, Fleisher Martin, Gersonde Rainer, Goldstein Steven L., Kuhn Gerhard, Mortyn P. Graham, Pahnke Katharina, Sachs Julian P. (2014). Biological response to millennial variability of dust and nutrient supply in the Subantarctic South Atlantic Ocean. Philosophical Transactions of the Royal Society A: Mathematical, Physical and Engineering Sciences.

[151] Lamy, F. et al. *Lithogenic mass accumulation rate of sediment core PS75/059-2*. PANGAEA hdoP (2014).

[152] Martínez-Garcia Alfredo, Rosell-Melé Antoni, Geibert Walter, Gersonde Rainer, Masqué Pere, Gaspari Vania, Barbante Carlo (2009). Links between iron supply, marine productivity, sea surface temperature, and CO2over the last 1.1 Ma. Paleoceanography.

[153] Kumar N (1995). Increased biological productivity and export production in the glacial Southern Ocean. Nature.

[154] Lamy F (2014). Increased dust deposition in the Pacific Southern ocean during glacial periods. Science.

[155] Kohfeld KE, Quéré CL, Harrison SP, Anderson RF (2005). Role of marine biology in glacial-interglacial CO2 cycles. Science.

[156] Martínez-Garcia A (2011). Southern Ocean dust–climate coupling over the past four million years. Nature.

[157] Herraiz-Borreguero L, Lannuzel D, van der Merwe P, Treverrow A, Pedro JB (2016). Large flux of iron from the Amery Ice Shelf marine ice to Prydz Bay, East Antarctica. J. Geophys. Res.: Oceans.

[158] Teitler, L., et al. Determination of Antarctic Ice Sheet stability over the last ∼500 ka through a study of iceberg-rafted debris. *Paleoceanography* **25**, 10.1029/2008PA001691 (2010).

[159] Diekmann B., Fütterer D. K., Grobe H., Hillenbrand C. D., Kuhn G., Michels K., Petschick R., Pirrung M. (2003). Terrigenous Sediment Supply in the Polar to Temperate South Atlantic: Land-Ocean Links of Environmental Changes during the Late Quaternary. The South Atlantic in the Late Quaternary.

[160] Manoj MC, Thamban M, Sahana A, Mohan R, Mahender K (2013). Provenance and temporal variability of ice rafted debris in the Indian sector of the Southern Ocean during the last 22,000 years. J. Earth Syst. Sci..

[161] Briggs RD, Pollard D, Tarasov L (2014). A data-constrained large ensemble analysis of Antarctic evolution since the Eemian. Quat. Sci. Rev..

[162] Weber S (2014). Millennial-scale variability in Antarctic ice-sheet discharge during the last deglaciation. Nature.

[163] Andrews JT (2000). Icebergs and iceberg rafted detritus (IRD) in the North Atlantic: facts and assumptions. Oceanography.

[164] Crémière A (2016). Timescales of methane seepage on the Norwegian margin following collapse of the Scandinavian Ice Sheet. Nat. Commun..

[165] Mackintosh AN (2014). Retreat history of the East Antarctic Ice Sheet since the Last Glacial Maximum. Quat. Sci. Rev..

[166] Pohlman JW (2017). Enhanced CO2 uptake at a shallow Arctic Ocean seep field overwhelms the positive warming potential of emitted methane. Proc. Natl Acad. Sci..

[167] Siegert Martin J., Priscu John C., Alekhina Irina A., Wadham Jemma L., Lyons W. Berry (2016). Antarctic subglacial lake exploration: first results and future plans. Philosophical Transactions of the Royal Society A: Mathematical, Physical and Engineering Sciences.

[168] Wadham JL (2012). Potential methane reservoirs beneath Antarctica. Nature.

[169] Yde JC (2010). Basal ice microbiology at the margin of the Greenland ice sheet. Ann. Glaciol..

[170] Booth AD (2012). Thin-layer effects in glaciological seismic amplitude-versus-angle (AVA) analysis: implications for characterising a subglacial till unit, Russell Glacier, West Greenland. Cryosphere.

[171] Dow CF (2013). Seismic evidence of mechanically weak sediments underlying Russell Glacier, West Greenland. Ann. Glaciol..

[172] Milkov AV (2004). Global estimates of hydrate-bound gas in marine sediments: how much is really out there?. Earth-Sci. Rev..

[173] Dickens GR (2011). Down the Rabbit Hole: toward appropriate discussion of methane release from gas hydrate systems during the Paleocene-Eocene thermal maximum and other past hyperthermal events. Clim. Past.

[174] Frank, M. & Mackensen, A. *Age model of sediment core PS2082-1*. PANGAEA (2002).

[175] Brathauer U, Abelmann A (1999). Age model of sediment core PS1778-8 In supplement to: Brathauer,1427 U; Abelmann, A (1999): Late Quaternary variations in sea surface temperatures and their1428 relationship to orbital forcing recorded in the Southern Ocean (Atlantic sector). Paleoceanography.

[176] Brathauer, U. Age model of sediment core PS1752-1. *PANGAEA*10.1594/PANGAEA.51757. (1997).

[177] Cook JM, Hodson AJ, Taggart AJ, Mernild SH, Tranter M (2017). A predictive model for the spectral “bioalbedo” of snow. J. Geophys. Res..

[178] Stibal Marek, Šabacká Marie, Žárský Jakub (2012). Biological processes on glacier and ice sheet surfaces. Nature Geoscience.

[179] Yallop ML (2012). Photophysiology and albedo-changing potential of the ice algal community on the surface of the Greenland ice sheet. ISME J..

[180] Uetake J, Naganuma T, Hebsgaard MB, Kanda H, Kohshima S (2010). Communities of algae and cyanobacteria on glaciers in west Greenland. Polar Sci..

[181] Benning LG, Anesio AM, Lutz S, Tranter M (2014). Biological impact on Greenland’s albedo. Nat. Geosci..

[182] Stibal M (2015). Different bulk and active bacterial communities in cryoconite from the margin and interior of the Greenland ice sheet. Environ. Microbiol. Rep..

[183] Lutz S, Anesio AM, Jorge Villar SE, Benning LG (2014). Variations of algal communities cause darkening of a Greenland glacier. FEMS Microbiol. Ecol..

[184] Williamson, C. J., et al. Ice algal bloom development on the surface of the Greenland Ice Sheet. *FEMS Microbiol. Ecol.* **94**, 10.1093/femsec/fiy025 (2018). 10.1093/femsec/fiy025 PMC6018781 29444265

[185] Hodson A (2010). The structure, biological activity and biogeochemistry of cryoconite aggregates upon an Arctic valley glacier: Longyearbreen, Svalbard. J. Glaciol..

[186] Lutz, S. et al. The biogeography of red snow microbiomes and their role in melting arctic glaciers. *Nat. Commun*. **7**, 10.1038/ncomms11968 (2016). 10.1038/ncomms11968 PMC4917964 27329445

[187] Wolff Eric W. (2013). Ice sheets and nitrogen. Philosophical Transactions of the Royal Society B: Biological Sciences.

[188] Telling, J., et al. Nitrogen fixation on Arctic glaciers, Svalbard. *J. Geophys. Res.-Biogeo.* **116**, 10.1029/2010jg001632 (2011).

[189] Bagshaw EA (2013). Do cryoconite holes have the potential to be significant sources of C, N, and P to downstream depauperate ecosystems of Taylor Valley, Antarctica?. Arct. Antarct. Alp. Res..

[190] Cook Joseph, Edwards Arwyn, Takeuchi Nozomu, Irvine-Fynn Tristram (2015). Cryoconite. Progress in Physical Geography: Earth and Environment.

[191] Hudson B (2014). MODIS observed increase in duration and spatial extent of sediment plumes in Greenland fjords. Cryosphere.

[192] Kump, L. R., Alley, R. B. Global chemical weathering on glacial timescales. In: *Material Fluxes on the Surface of the Earth* . (The National Academies Press, Washington D.C., U.S.A. 1994).

[193] Vance D, Teagle DAH, Foster GL (2009). Variable quaternary chemical weathering fluxes and imbalances in marine geochemical budgets. Nature.

[194] Foster GL, Vance D (2006). Negligible glacial-interglacial variation in continental chemical weathering rates. Nature.

[195] Graly JA, Drever JI, Humphrey NF (2017). Calculating the balance between atmospheric CO2 drawdown and organic carbon oxidation in subglacial hydrochemical systems. Glob. Biogeochem. Cycle.

[196] Graly JA, Humphrey NF, Landowski CM, Harper JT (2014). Chemical weathering under the Greenland Ice Sheet. Geology.

[197] Boyd ES, Hamilton TL, Havig JR, Skidmore ML, Shock EL (2014). Chemolithotrophic primary production in a subglacial ecosystem. Appl. Environ. Micro..

[198] Boyd ES (2011). Diversity, abundance, and potential activity of nitrifying and nitrate-reducing microbial assemblages in a subglacial ecosystem. Appl. Environ. Micro..

