# Publisher Correction: Ice sheets matter for the global carbon cycle

**DOI:** 10.1038/s41467-019-11997-x

**Published:** 2019-09-09

**Authors:** J. L. Wadham, J. R. Hawkings, L. Tarasov, L. J. Gregoire, R. G. M. Spencer, M. Gutjahr, A. Ridgwell, K. E. Kohfeld

**Affiliations:** 1 University of Bristol, Bristol, BS8 1TH UK; 2 National High Magnetic Field Lab and Earth, Ocean and Atmospheric Sciences, Florida State University, Tallahassee, FL 32306 USA; 3 German Research Centre for Geosciences GFZ, 14473 Potsdam, Germany; 4 Memorial University, St. John’s, NF A1B 3X9 Canada; 5 University of Leeds, Leeds, LS6 1AN UK; 6 GEOMAR, 24148 Kiel, Germany; 7 University of California, Riverside, CA 94720 USA; 8 Simon Fraser University, Burnaby, BC 8888 Canada

**Keywords:** Cryospheric science, Biogeochemistry, Carbon cycle

Correction to: *Nature Communications* 10.1038/s41467-019-11394-4, published online 15 August 2019.

The original version of this Article contained an error in Fig. 5, in which the grey line representing ‘EPICA Dust’ was inadvertently omitted from Fig. 5h. This has been corrected in both the PDF and HTML versions of the Article.

