# Supplementary material for: Ice sheets matter for the global carbon cycle
Source: Nat Commun. 2019 Aug 15;10:3567. doi: 10.1038/s41467-019-11394-4 (PMC6695407; doi:10.1038/s41467-019-11394-4)
Supplement: Supplementary file 1 — Supplementary Information [file 41467_2019_11394_MOESM1_ESM.pdf]

Supplementary Information

To accompany the Review Article "Ice sheets matter for the global carbon cycle" by J.L. Wadham, J. R. Hawkings, L. Tarasov, L. Gregoire, R. G.M. Spencer, M. Gutjahr, A. Ridgwell and K. Kohfeld

Supplementary Figures

Supplementary Figure 1. Time series of deglacial meltwater melt fluxes separated into subglacial meltwater, runoff and iceberg fluxes from northern hemisphere ice sheets (North American ice sheet complex, Eurasian, Greenland) from the 25 kyr B.P. to the present day. HS = Heinrich Stadial, MP = Meltwater Pulse.

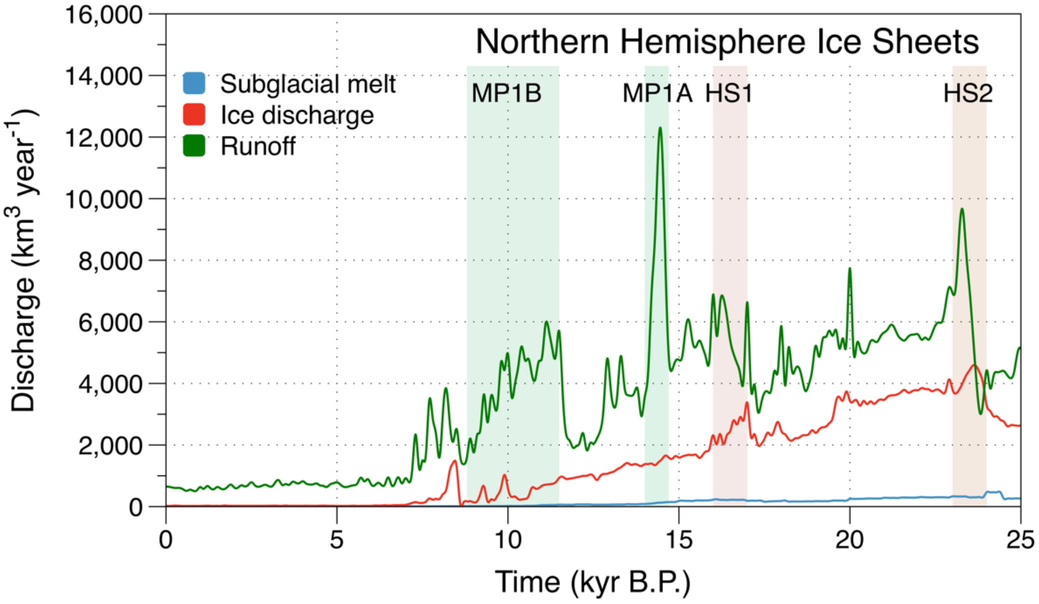

Supplementary Figure 2. Map indicating the locations of sub-Antarctic marine cores presented in Figure 5 (main manuscript), along with the location of the EPICA Dome C core, the present day Polar Front position and the estimated LGM ice sheet extent.

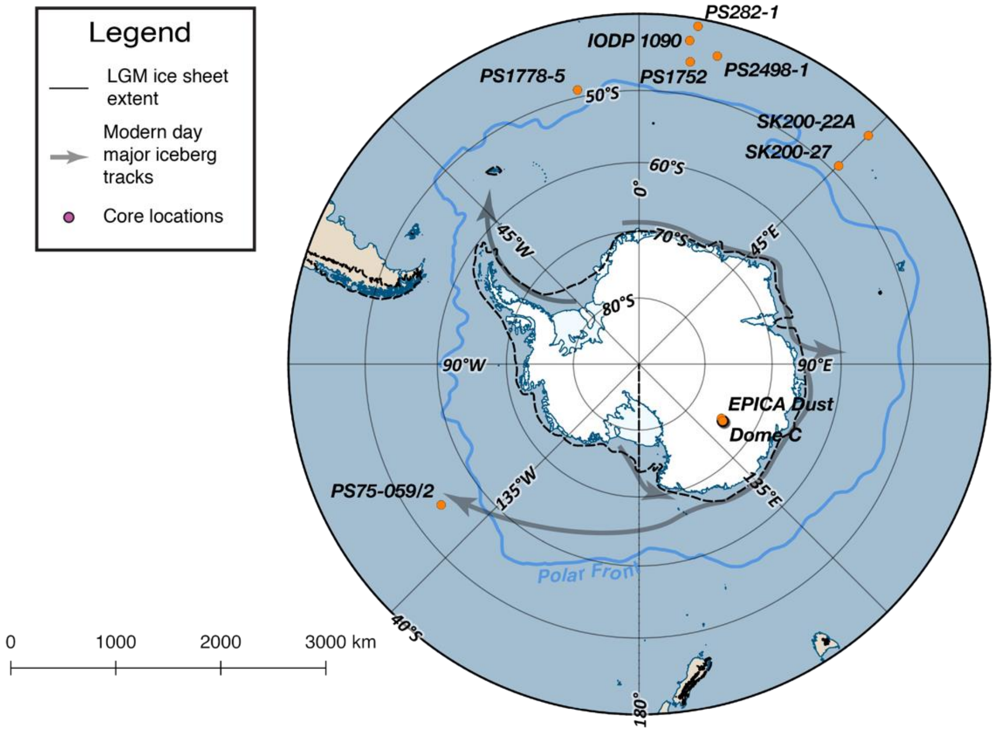

Supplementary Tables

Supplementary Table 1. Estimated dissolved concentrations of nutrients and organic carbon associated with Greenland and Antarctic freshwater fluxes (all values to 3/4 sig. figs).

Concentrations Dissolved Species - Northern Hemisphere (Greenland)						
	RUNOFF			ICEBERGS		
	min	mid	max	min	mid	max
FW Flux (km^3 a^{-1})	404	545	728	494	498	501
DIN (∝[illegible])	0.500	1.50	7.50	0.500	1.40	2.30
DIP (∝M)	0.160	0.230	0.300	n/a	n/a	n/a
DFe (∝M)	0.232	0.706	4.74	n/a	n/a	n/a
DSi (∝M)	0.800	9.60	41.4	n/a	n/a	n/a
DOC (∝M)	6.46	29.6	97.2	0.833	5.83	15.8

Concentrations Particulate Species - Northern Hemisphere (Greenland)						
	RUNOFF			ICEBERGS		
	min	mid	max	min	mid	max
Sediment Flux (Tg a^{-1})	260	604	2822	138	249	401
PIN (∝M-runoff, % bergs)	0.300	1.40	3.70	0.0000110	0.000150	0.00389
PIP (%)	0.000710	0.00151	0.00072	0.000190	0.000690	0.00120
PFe (%)	0.110	0.150	0.180	0.0300	0.0760	0.194
PSi (%)	0.510	0.910	1.21	0.160	0.280	0.470
POC (%)	0.0250	0.0675	0.132	0.185	0.440	0.660

Supplementary Table 2 Estimated sediment-bound concentrations of nutrients and organic carbon associated with Greenland and Antarctic freshwater fluxes (all values to 3/4 sig. figs).

**Concentrations Dissolved Species - Southern Hemisphere (Antarctica)**						
	**RUNOFF**			**ICEBERGS**		
	min	mid	max	min	mid	max
FW Flux (km^3 a^{-1})	41.0	65.0	79.3	1231	1562	1893
DIN (∝[illegible])	2.10	3.30	4.50	0.375	2.93	5.54
DIP (∝M)	2.40	3.10	3.80	n/a	n/a	n/a
DFe (∝M)	0.470	4.70	47.0	n/a	n/a	n/a
DSi (∝M)	130	140	210	n/a	n/a	n/a
DOC (∝M)	166	221	276	0.833	25.0	71.7
**Concentrations Particulate Species - Southern Hemisphere (Antarctica)**						
	**RUNOFF**			**ICEBERGS**		
	min	mid	max	min	mid	max
Sediment Flux (Tg a^{-1})	26	72	307	345	781	1514
PIN (∝M-runoff, % bergs)	0.300	1.400	3.70	0.0000110	0.000150	0.00389
PIP (%)	0.000710	0.00151	0.00072	0.000190	0.000690	0.00120
PFe (%)	0.110	0.150	0.180	0.0300	0.076	0.194
PSi (%)	0.510	0.910	1.21	0.160	0.280	0.470
POC (%)	0.100	0.461	1.00	0.100	0.461	1.00

Supplementary Table 3 Fluxes of nutrients and organic carbon associated with freshwater fluxes from present day (Greenland, Antarctic) and former (northern hemisphere only) ice sheets. All values quoted to 3 sig. figs.

**PRESENT DAY**														
**Northern Hemisphere (Greenland)**														
	Fluxes (Gg a^{-1})			*Fluxes (Gg a^{-1})*			*Fluxes (Gg a^{-1})*				*Fluxes (Tg a^{-1})*			
	min	mid	max	min	mid	max	min	mid	max		*Runoff*	*Icebergs*	*Total*	*Upwelling E Greenland*
	Runoff - dissolved			***Runoff - SPM***			***Icebergs***			***TOTAL (mid)***				*Cape et al (2019)*
N	2.83	11.4	57.2	1.45	9.16	32.3	3.52	10.2	31.7		0.0206	0.0102	0.0308	0.012-0.040
P	2.00	3.88	6.76	1.84	9.13	20.3	0.263	1.72	4.81		0.0130	0.0017	0.0147	0.002 - 0.009
	Fluxes (Tg a^{-1})			*Fluxes (Tg a^{-1})*			*Fluxes (Tg a^{-1})*				*Fluxes (Tg a^{-1})*			
Fe	0.00523	0.0215	0.193	0.286	0.907	5.08	0.0415	0.189	0.778		0.928	0.189	1.12	n.d.
Si	0.00905	0.146	0.844	1.32	5.50	34.1	0.221	0.697	1.88		5.65	0.697	6.34	0.014-0.066
OC	0.0313	0.193	0.849	0.0649	0.408	3.72	0.261	1.13	2.74		0.601	1.13	1.73	
**Southern Hemisphere**														
	min	mid	max	min	mid	max	min	mid	max					
	Runoff - dissolved			***Runoff - SPM***			***Icebergs***			***TOTAL (mid)***				
	Fluxes (Gg a^{-1})			*Fluxes (Gg a^{-1})*			*Fluxes (Gg a^{-1})*				*Fluxes (Tg a^{-1})*			
N	1.21	3.00	5.00	0.172	1.27	4.11	6.50	65.2	147		0.00428	0.0652	0.0695	
P	1.77	3.63	5.42	0.187	1.09	2.21	0.65	5.39	18.2		0.00472	0.00539	0.0101	
	Fluxes (Tg a^{-1})			*Fluxes (Tg a^{-1})*			*Fluxes (Tg a^{-1})*				*Fluxes (Tg a^{-1})*			
Fe	0.00108	0.0171	0.208	0.0290	0.108	0.55	0.103	0.594	2.94		0.125	0.594	0.719	
Si	0.149	0.255	0.466	0.134	0.656	3.7	0.551	2.19	7.12		0.911	2.19	3.10	
OC	0.0817	0.172	0.263	0.0264	0.332	3.1	0.71	4.07	15.7		0.505	4.07	4.57	
**Peak Deglaciation (14.5 kyr B.P.)**														
**Northern Hemisphere (Greenland, Laurentide, European)**														
	min	mid	max	min	mid	max	min	mid	max					
	Runoff - dissolved			***Runoff - SPM***			***Icebergs***			***TOTAL (mid)***				
N	61.9	251	1600	31.8	200	708	10.9	31.6	98.2		0.451	0.032	0.483	
P	43.8	85.0	148	40.4	200	1850	0.81	5.32	14.9		0.285	0.005	0.290	
	Fluxes (Tg a^{-1})			*Fluxes (Tg a^{-1})*			*Fluxes (Tg a^{-1})*				*Fluxes (Tg a^{-1})*			
Fe	0.115	0.47	4.22	6.26	19.8	111	0.128	0.586	2.41		20.3	0.586	20.9	
Si	0.198	3.21	18.5	29.0	120	748	0.685	2.16	5.83		124	2.16	126	
OC	0.686	4.23	18.6	1.42	8.93	81.5	0.81	3.50	8.49		13.2	3.50	16.7	

Supplementary Methods

Laboratory Methods - nutrient concentrations in Greenland icebergs

Concentrations of inorganic nitrogen (extractable NO_3^{-1} and NH_4^+) loosely bound to iceberg entrained sediment were determined from 11 Greenland icebergs samples collected from Tunulliarfik Fjord, Greenland (61.1°N 45.4°W) in July 2013, using 2.0 M KCl[1]. These extractions gave mean concentrations of 1.5 μg N g^{-1} (range 0.11-38.9 μg N g^{-1}). Loosely-bound P ($MgCl_2$-extractable) and Fe-/Al-bound phosphorous (NaOH-extractable) concentrations associated with sediments in icebergs were determined from 15 Greenland icebergs sampled from Tunulliarfik Fjord in July 2013 (as with exchangeable nitrogen species above)[2]. Both these P phases are taken to be bioavailable, and gave combined mean concentrations of 6.9 μg P g^{-1} (range 1.9-12 μg P g^{-1}).

Modelled freshwater fluxes over the last 25 kyr (Northern Hemisphere)

To calculate freshwater fluxes from former northern hemisphere ice sheets, we employed the GLAC-1D model, published in refs [3,4,5], and used for PMIP4[6]. Tarasov et al. computed a posterior probability distribution for the deglacial evolution of the North American ice sheet complex by large ensemble Bayesian calibration of the 3D Glacial Systems Model (GSM). In short, 39 ensemble GSM parameters (attempting to account for uncertainties in climate forcing, basal drag, ice calving, and marginal surface mass-balance/extent) were calibrated against a large set of geophysical (relative sea level and present day vertical velocities) and geological (ice margin extent and strandline records of proglacial lake extent) constraints. This model was also used to compute freshwater flux time series for all northern hemisphere ice sheets (Greenland, North American ice sheet complex, European ice sheets), subsequently used to calculate past fluxes of nutrients and organic carbon (see Supplementary Figure 1). Freshwater flux terms can be sub-divided into subglacial meltwater (generated by geothermal and strain heating of basal ice layers within the ice sheet), runoff (subglacially routed) and ice discharge due to iceberg calving.

GLAC-1D replicates key features associated with the decreasing northern hemisphere ice volumes over the last deglaciation (Supplementary Figure 1). Peak northern hemisphere ice discharge via iceberg calving was apparent at the Last Glacial Maximum (LGM, c. 24-25 kyr B.P.), which is consistent with the presence of large ice sheets (and the accompanying extensive marine exposure of their margins) with a predominance of warm-basal conditions and high ice stream activity[7]. Modelled runoff fluxes from northern hemisphere ice sheets rose from the LGM onwards, and meltwater pulse events 1A at 14.5 kyr and 1B at c. 11 kyr are relatively well resolved in Glac1D, as is the fall to low freshwater fluxes by 7 kyr B.P. Temporal trends in modelled freshwater fluxes compared well with previous work[8]. Subglacial melt fluxes were generally low, peaking around the LGM.

Freshwater fluxes were not computed for the Antarctic Ice Sheet because of sensitivity to poorly constrained precipitation in the model. Hence, we rely on previously published values to estimate nutrient fluxes from the Antarctic Ice Sheet at the present day/past[9].

Calculations of ice sheet nutrient and carbon fluxes from northern and southern hemisphere ice sheets

We present an envelope of what we consider to be bioavailable fluxes of nutrients, together with total organic carbon, from past (northern hemisphere) and present-day ice sheets (northern and southern hemisphere). Bioavailable dissolved fluxes include: Dissolved Inorganic Nitrogen (DIN), Dissolved Inorganic Phosphorus (DIP), Dissolved Silica (DSi) and Dissolved Fe (DFe, comprising filterable iron <45µm) and Dissolved Organic Carbon (DOC). Bioavailable nutrient fluxes associated with suspended sediment (SS) in glacial runoff include loosely-bound N (PN), loosely-bound P + NaOH-P (PP)[2], labile Fe associated with ascorbate-extractable iron oxyhydroxide nanoparticles[11] (PFe) and amorphous Si (PSi)[10]. Particulate organic carbon fluxes (POC) associated with suspended sediments (SS) in runoff were also computed.

Nutrient and organic carbon fluxes for present day ice sheets are computed from the product of the concentration (min, max and mid-range values) of the nutrient species and the freshwater or sediment flux (also using min, max and mid-range values). We also present peak potential nutrient fluxes for northern hemisphere ice sheets, at 14.5 kyr B.P., which corresponded to Meltwater Pulse 1a (Supplementary Figure 1). Freshwater fluxes at this time were taken from simulations using the GLAC1-D model[3,4,5] (see Supplementary Figure 1). To calculate peak nutrient and organic carbon fluxes for northern hemisphere ice sheet runoff and icebergs we applied scaling factors to present day nutrient fluxes, assuming that the freshwater flux changes, but the nutrient concentration associated with this flux remains the same. The scaling factor was 22 for northern hemisphere runoff fluxes, assuming a northern hemisphere ice sheet runoff flux of 545 $km^3\ a^{-1}$ [12] at present (Greenland Ice Sheet) and 11,932 km^3 at 14.5 kyr B.P. (Greenland Ice Sheet and European/North American ice sheet complexes Supplementary Figure 1). For icebergs, the scaling factor was 3.1, assuming a present day ice discharge of 498 $km^3\ a^{-1}$ [12] and peak deglaciation fluxes of 1542 $km^3\ a^{-1}$ (Supplementary Figure 1). We assume that the proportional uncertainty around these peak freshwater flux values is similar to the present day.

Summarised concentrations and fluxes of nutrients are organic carbon are presented in Supplementary Tables 1-3. Details of how these were calculated are given in the next section.

Northern Hemisphere ice sheet nutrient and organic carbon fluxes

Glacial runoff

The Greenland Ice Sheet is the only ice sheet in the northern hemisphere at the present day. We employ previously published concentrations (min, max, mid-range) of DIN/PN[13], DIP/PP[2], Fe/PFe[14], Si/PSi[10] and DOC/POC[15] in subglacially routed runoff from the Greenland Ice Sheet. Freshwater fluxes are taken from ref. [12] and include minimum, mean and maximum values for the period 2011-2016. For fluxes of nutrients and organic carbon associated with SS in runoff, we take SS concentrations from ref. [14] (1.1 g L^{-1}, range = 0.64-3.9 g L^{-1}). This gives typical SS fluxes of 604 Tg a^{-1} (range = 260-2822 Tg a^{-1}).

Icebergs

Particulate nutrient and organic carbon fluxes associated with Greenland icebergs at the present day were calculated assuming iceberg debris concentrations of 0.5 g L^{-1} (range = 0.28-0.8 g L^{-1})[16] and annual iceberg freshwater fluxes of 498 km^3 a^{-1} (range = 494-501) km^3 a^{-1} [12]. Thus, sediment fluxes associated with Greenland icebergs were 249 Tg a^{-1} (range = 138-401) Tg a^{-1}.

Dissolved fluxes (DIN and DOC only) associated with Greenland icebergs were calculated from the product of the freshwater flux and an estimate of the dissolved species concentration. DIN concentrations associated with Greenland icebergs were taken from ref. [13] (min and max concentrations measured in surface Greenland ice) and ref. [17] (mean concentrations in Greenland ice cores). For DOC concentrations in icebergs, we employ minimum, mean and maximum concentrations of DOC in Greenlandic ice reported in ref. [18]. DIP, DFe and DSi concentrations associated with Greenland icebergs were considered to be negligible compared with particulate fluxes based on measured their concentrations in glacial ice and meltwaters[10,19,20].

Particulate fluxes of nutrients associated with Greenland icebergs were derived from the iceberg rafted debris flux and the estimated nutrient concentration associated with those sediments. Concentrations of loosely-bound N associated with iceberg entombed sediment from Tunulliarfik Fjord (Greenland), were 1.5 ng g^{-1} (0.1 – 38.9 ng g^{-1} see first section). Loosely-bound P and NaOH-P concentrations associated with sediments entombed in icebergs in icebergs sampled from Tunulliarfik Fjord (see first section) were 1.8 (0.6-3.1) ng g^{-1} and 5.1 (1.3-8.9) ng g^{-1} respectively, the sum of which is considered to be equal to the bioavailable concentration. Bioavailable (ascorbic acid extractable) Fe fluxes associated with sediments hosted in Greenland icebergs at the present day were calculated using minimum, mean and maximum concentrations reported in ref. [21]. PSi concentrations associated with iceberg rafted debris were taken from measured mean, minimum and maximum concentrations of amorphous Si associated with Greenland icebergs[10]. We employed POC concentrations reported for debris entombed in basal ice

sampled from the ice sheet margins to calculate POC fluxes from icebergs, giving a mid-range concentration of 0.31 % (range 0.185 % - 0.44 %)[22,23].

Southern Hemisphere ice sheet nutrient and organic carbon fluxes

Subglacial meltwater

There is much greater uncertainty around Antarctic subglacial meltwater nutrient and organic carbon fluxes due to very limited data. We employed modelled estimations of the subglacial meltwater flux derived from ref. [9] (65 km^3 a^{-1}, range = 41-79 km^3 a^{-1} [9]). For sediment-bound nutrients and in the absence of Antarctic data, we assumed mean suspended sediment concentrations in Antarctic subglacial meltwater similar to Greenland (1.1 g L^{-1}, range = 0.63-3.88 g L^{-1}), giving a sediment flux of 72 Tg a^{-1} (range = 26-307 Tg a^{-1}). DIN, DIP, DSi and DOC concentrations (mid-range, max, min) were derived from concentrations reported from Subglacial Lake Whillans[24,25]. A mid-range DFe concentration for Antarctic subglacial meltwaters was taken from maximum values reported for Greenland Ice Sheet runoff [14], with minimum and maximum values calculated as 10x less or 10x greater than the mid-range value. This assumes that meltwater residence times would likely be enhanced beneath the Antarctic Ice Sheet compared with Greenland[26]. In the absence of data on the concentrations of nutrients associated with suspended sediment in Antarctic subglacial meltwater, we employed estimates of PN, PP, PSi and PFe concentrations in SS associated with Greenland subglacial runoff[2,10,13,14]. For OC concentrations associated with suspended sediments in Antarctic subglacial meltwater, we employed the POC concentrations reported in surface lake sediments in Subglacial Lake Whillans to give a mid-range value of 0.46 %[24] and assumed minimum (0.1 %) and maximum (1%) values in line with concentrations measured from IODP cores from around Antarctica[27].

Icebergs

Present day nutrient and organic carbon fluxes associated with Antarctic icebergs were calculated using published estimates of the freshwater flux (minimum =1321 km^3 [28], maximum = 1893 km^3 [29], with mid-range calculated as the mean of the minimum and maximum values = 1562 km^3), mid-range, minimum and maximum estimates of the iceberg sediment concentration[16] and estimates of nutrient/organic carbon concentration in the ice (DIN and DOC only) and/or associated with iceberg entombed debris (PN, PP, PFe, PSi, POC). We assumed sediment concentrations of 0.28, 0.5 and 0.8 g L^{-1} in Antarctic icebergs[16], yielding iceberg rafted debris fluxes of 345, 781 and 1514 Tg a^{-1} respectively.

Dissolved nutrient and organic carbon fluxes associated with Antarctic icebergs were calculated for DIN and DOC, from the product of the freshwater flux and estimated dissolved concentration in the ice. Mean DIN concentrations in Antarctic icebergs were assumed to be similar to mean DIN in Antarctic ice cores[17], with minimum and maximum values taken from measured concentrations of nitrate[30] and

ammonium[31] in the EPICA Dome C ice core, Antarctica. DOC concentrations in icebergs were assumed to be similar to those reported for englacial Antarctic ice[18]. DIP, DFe and DSi fluxes associated with Antarctic icebergs were assumed to be negligible, since their concentrations in ice are generally low[32,33].

Particulate nutrient and organic carbon fluxes were derived from the sediment flux associated with Antarctic icebergs and the nutrient concentration associated with those sediments. In the absence of bioavailable N and P concentrations associated with Antarctic iceberg rafted debris, we employ loosely-bound N/P and NaOH- P concentrations reported in this manuscript for Greenland icebergs (see first section). Bioavailable (ascorbate extractable) Fe concentrations associated with iceberg entombed debris were taken directly from ref. [21], which reports typical concentrations for Antarctic bergs. PSi concentrations of iceberg rafted debris were also assumed to be similar to those observed for Greenland Ice Sheet icebergs, in the absence of Antarctic data[10]. We assumed that concentrations of POC associated with iceberg entombed debris ranged from 0.1 % to 1% (typical minimum and mean Antarctic marine sediment concentrations)[34], with a mid-range value of 0.46 % (Subglacial Lake Whillans concentrations[24]).

A summary of the freshwater/sediment fluxes and concentrations of nutrients and organic carbon associated with runoff and icebergs in both hemispheres can be found in Supplementary Tables 1, 2 and 3.

Supplementary References

1. Maynard DG, Kalra YP, Crumgaugh JA. Nitrate and exchangeable ammonium nitrogen. In: *Soil Sampling and Methods of Analysis* (ed^(eds Carter MR, Gregorich EG). CRC Press (2007).

2. Hawkings J, *et al.* The Greenland Ice Sheet as a hot spot of phosphorus weathering and export in the Arctic. *Global Biogeochem Cy* **30**, 191-210, (2016).

3. Briggs RD, Pollard D, Tarasov L. A data-constrained large ensemble analysis of Antarctic evolution since the Eemian. *Quaternary Sci Rev* **103**, 91-115, (2014).

4. Tarasov L, Dyke AS, Neal RM, Peltier WR. A data-calibrated distribution of deglacial chronologies for the North American ice complex from glaciological modeling. *Earth Planet Sc Lett* **315–316**, 30-40, (2012).

5. Tarasov L, Richard Peltier W. Greenland glacial history and local geodynamic consequences. *Geophys J Int* **150**, 198-229, 10.1046/j.1365-246X.2002.01702.x (2002).

6. Ivanovic RF, *et al.* Transient climate simulations of the deglaciation 21–9 thousand years before present (version 1) – PMIP4 Core experiment design and boundary conditions. *Geosci Model Dev* **9**, 2563-2587, 10.5194/gmd-9-2563-2016 (2016).

7. Marshall S, Clarke PU. Basal temperature evolution of North American Ice Sheets and implications for the 100 kyr cycle. *Geophys Res Lett* **29(24)**, 2214, doi:10.1029/2002GL015192 (2002).

8. Tranter M, *et al.* Direct effect of ice sheets on terrestrial bicarbonate, sulphate and base cation fluxes during the last glacial cycle: minimal impact on atmospheric CO2 concentrations. *Chem Geol* **190**, 33-44, Pii S0009-2541(02)00109-2 (2002).

9. Pattyn F. Antarctic subglacial conditions inferred from a hybrid ice sheet/ice stream model. *Earth Planet Sc Lett* **295**, 451-461, DOI 10.1016/j.epsl.2010.04.025 (2010).

10. Hawkings J, *et al.* Ice sheets as a missing source of silica to the world's oceans. *Nature Communications* **8**, Doi:10.1038/ncomms14198, DOI:10.1038/ncomms14198 (2017).

11. Raiswell R, *et al.* Contributions from glacially derived sediment to the global iron (oxyhydr)oxide cycle: Implications for iron delivery to the oceans. *Geochim Cosmochim Ac* **70**, 2765-2780, DOI 10.1016/j.gca.2005.12.027 (2006).

12. Bamber JL, *et al.* Land Ice Freshwater Budget of the Arctic and North Atlantic Oceans: 1. Data, Methods, and Results. *Journal of Geophysical Research: Oceans* **123**, 1827-1837, 10.1002/2017JC013605 (2018).
13. Wadham JL, *et al.* Sources, cycling and export of nitrogen on the Greenland Ice Sheet. *Biogeosciences Discuss*, 6339-6352, (2016).

14. Hawkings J, *et al.* Ice sheets as a significant source of highly reactive nanoparticulate iron to the oceans. *Nature Communications* **5**, Doi:10.1038/ncomms4929, DOI:10.1038/ncomms4929 (2014).
15. Lawson E, *et al.* Greenland Ice Sheets exports labile organic carbon to the Arctic oceans. *Biogeosciences Discussions* **10**, 19311–19345, (2013).

16. Death R, *et al.* Antarctic ice sheet fertilises the Southern Ocean. *Biogeosciences* **11**, 2635-2643, 10.5194/bg-11-2635-2014 (2014).

17. Wolff EW. Ice sheets and nitrogen. *Philos T R Soc B* **368**, Doi 10.1098/Rstb.2013.0127 (2013).

18. Hood E, Battin TJ, Fellman J, O'Neel S, Spencer RGM. Storage and release of organic carbon from glaciers and ice sheets. *Nature Geosci* **8**, 91-96, 10.1038/ngeo2331(2015).

19. Bhatia MP, Kujawinski EB, Das SB, Breier CF, Henderson PB, Charette MA. Greenland meltwater as a significant and potentially bioavailable source of iron to the ocean (vol 6, pg 274, 2013). *Nat Geosci* **6**, 503-503, Doi 10.1038/Ngeo1833 (2013).

20. Kjaer HA, *et al.* Greenland ice cores constrain glacial atmospheric fluxes of phosphorus. *Journal of Geophysical Research - Atmospheres* **120**, 10,810–810,822, (2015).

21. Raiswell R, *et al.* Potentially Bioavailable Iron Delivery by Iceberg hosted Sediments and Atmospheric Dust to the Polar Oceans. *Biogeosciences Discuss*, Doi:10.5194/bg-2016-5120, doi:10.5194/bg-2016-20 (2016).

22. Stibal M, *et al.* Methanogenic potential of Arctic and Antarctic subglacial environments with contrasting organic carbon sources. *Global Change Biology* **18**, 3332-3345, 10.1111/j.1365-2486.2012.02763.x (2012).

23. Yde JC, *et al.* Basal ice microbiology at the margin of the Greenland ice sheet. *Annals of Glaciology* **51**, 71-79, 10.3189/172756411795931976 (2010).

24. Christner BC, *et al.* A microbial ecosystem beneath the West Antarctic ice sheet. *Nature* **512**, 310-313, 10.1038/nature13667 (2014).

25. Michaud AB, *et al.* Solute sources and geochemical processes in Subglacial Lake Whillans, West Antarctica. *Geology*, Doi:10.1130/g37639.37631, 10.1130/g37639.1 (2016).

26. Wadham JL, *et al.* Biogeochemical weathering under ice: Size matters. *Global Biogeochem Cycles* **24**, Doi:10.1029/2009gb003688, 10.1029/2009gb003688 (2010).

27. Wadham JL, *et al.* The potential role of the Antarctic Ice Sheet in global biogeochemical cycles. *Earth and Environmental Science Transactions of the Royal Society of Edinburgh* **104**, 55-67, 10.1017/S1755691013000108 (2013).

28. Depoorter MA, *et al.* Calving fluxes and basal melt rates of Antarctic ice shelves. *Nature* **502**, Doi:10.1038/Nature12567, Doi.10.1038/Nature12567 (2013).

29. Rignot E, Mouginot J, Scheuchl B, van den Broeke M, van Wessem MJ, Morlighem M. Four decades of Antarctic Ice Sheet mass balance from 1979–2017. *Proceedings of the National Academy of Sciences* **116**, 1095, 10.1073/pnas.1812883116 (2019).

30. Wolff EW, *et al.* Changes in environment over the last 800,000 years from chemical analysis of the EPICA Dome C ice core. *Quaternary Sci Rev* **29**, 285-295, (2010).

31. Kaufmann P, *et al.* Ammonium and non-sea salt sulfate in the EPICA ice cores as indicator of biological activity in the Southern Ocean. *Quaternary Sci Rev* **29**, 313-323, (2010).

32. Kim I, Kim G, Choy EJ. The significant inputs of trace elements and rare earth elements from melting glaciers in Antarctic coastal waters. *Polar Res* **34**, Doi:10.3402/polar.v3434.24289, 10.3402/polar.v34.24289 (2015).

33. Raiswell R, Hawkings J, Elsenousy A, Death R, Tranter M, Wadham J. Iron in Glacial Systems: Speciation, Reactivity, Freezing Behavior, and Alteration During Transport. *Frontiers in Earth Science* **6**, 222, (2018).

34. Wadham JL, *et al.* Potential methane reservoirs beneath Antarctica. *Nature* **488**, 633-637, 10.1038/nature11374 (2012).
